# Association of oral health with all-cause and cause-specific mortality in older Chinese adults: A 14-year follow-up of the Guangzhou Biobank Cohort study

**DOI:** 10.7189/jogh.14.04111

**Published:** 2024-07-05

**Authors:** Bai Jing Zhou, Chao Qiang Jiang, Ya Li Jin, Shiu Lun Au Yeung, Tai Hing Lam, Kar Keung Cheng, Wei Sen Zhang, Lin Xu

**Affiliations:** 1 School of Public Health, Sun Yat-sen University, Guangzhou, Guangdong, China; 2 Molecular Epidemiology Research Center, Guangzhou Twelfth People’s Hospital, Guangzhou, Guangdong, China; 3 School of Public Health, The University of Hong Kong, Hong Kong, China; 4 Institute of Applied Health Research, University of Birmingham, Birmingham, UK; 5 Great Bay Area Public Health Research Collaboration, Guangdong-Hong Kong- Macao, China

## Abstract

**Background:**

Poor oral hygiene is associated with overall wellness, but evidence regarding associations of oral health with all-cause mortality remain inconclusive. We aimed to examine the associations of oral health with all-cause and cause-specific mortality in middle-aged and older Chinese adults.

**Methods:**

28 006 participants were recruited from 2003–2008 and followed up until 2021. Oral health was assessed by face-to-face interview and causes of death was identified via record linkage. Cox regression yielded hazard ratios (HRs) and 95% confidence intervals (CIs) with adjustment of multiple potential confounders.

**Results:**

During an average of 14.3 years of follow-up, we found that a lower frequency of toothbrushing was associated with higher risks of all-cause mortality with a dose-response pattern (*P* for trend <0.001). Specially, the adjusted HR (95% CI) (vs. ≥ twice/d) was 1.16 (1.10, 1.22) (*P* < 0.001) for brushing once/d and 1.27 (1.00, 1.61) (*P* = 0.048) for < once/d. Similar associations were also found for cardiovascular disease (CVD), stroke, and respiratory disease mortality, but not for ischemic heart disease (IHD) and cancer mortality. A greater number of missing teeth was also associated with higher risks of all-cause, CVD, stroke, and respiratory disease mortality with a dose-response pattern (all *P* for trend <0.05). The association of missing teeth with all-cause mortality was stronger in lower-educated participants.

**Conclusions:**

Both less frequent toothbrushing and a greater number of missing teeth were associated with higher risks of all-cause, CVD, stroke, and respiratory disease mortality, showing dose-response patterns, but not with IHD and cancer mortality. Moreover, the dose-response association of missing teeth with all-cause mortality was stronger in lower-educated participants.

The Global Burden of Disease (GBD) study estimated 3.5 billion individuals worldwide experienced compromised oral health encompassing dental caries, severe periodontitis, tooth loss, edentulism (complete tooth loss) and other oral conditions in 2017 [1,2]. Moreover, the age-standardised prevalence of severe periodontitis increased by 8.44% from 1990 to 2019 [3]. Despite being largely preventable, oral diseases persist with high prevalence and growly inadequate treatment, particularly in less developed countries.

The Fourth National Oral Health Survey in China showed that about 90% of Chinese adults suffered from periodontal disease in 2015–2016, with severity strongly associated with age [4]. However, only less than 20% of Chinese adults were aware of periodontal prevention and treatment strategies [5]. Given the high prevalence but limited recognition of periodontal disease, scientific evidence on the health impact of poor oral health is needed to advocate for improved oral health awareness, oral hygiene practices, and public health and clinical dental care.

Poor oral health, including less frequent toothbrushing and tooth loss, has been shown to be prospectively associated with various non-communicable diseases including cardiovascular disease (CVD) [6], type 2 diabetes [7], respiratory disease [8], and cancer [9], which may increase the mortality risk. However, evidence regarding associations of poor oral health with all-cause and cause-specific mortality risk has been less definitive. A systematic review with meta-analysis of 48 cohort studies showed that both periodontitis and edentulism were significantly associated with higher risks of all-cause, CVD and cancer mortality, while significant risk of pneumonia mortality was observed in for edentulism [10]. Furthermore, the number of missing teeth and risks of respiratory [11,12] and cancer mortality [11,13,14] remained inconclusive. Notably, previous studies predominantly focused on Western or developed countries, resulting in limited evidence and awareness of the health implications of poor oral health in non-white and less-developed populations, particularly in China. Two cohort studies on 0.5 million Chinese adults showed that infrequent toothbrushing was associated with higher risks of vascular events and certain cancers, but neither of them investigated specified toothbrushing frequency and dose-response patterns [8,15]. Four previous studies reported the associations between tooth loss and mortality risk specifically in Chinese populations, but the results for all-cause, CVD, and respiratory mortality risk were controversial [16–19]. In addition, two of them were based on periodontal patients [18,19], but not community-based, which may limit the generalisability of the results.

Hence, using data from a well-established population-based cohort, the Guangzhou Biobank Cohort Study (GBCS), we examined the association of oral health status, including toothbrushing, gums bleeding and tooth loss with all-cause and cause-specific mortality in 28 006 older Chinese adults who were recruited in 2003–2008 and followed up until April 2021.

## METHODS

### Study participants

All participants were from the Guangzhou Biobank Cohort Study (GBCS). Details of study design, characteristics of study participants, and some prospective study results in GBCS have been reported previously [20]. Briefly, GBCS is a three-way collaborative project among Guangzhou Twelfth People’s Hospital, the Universities of Hong Kong, China and Birmingham, UK. Participants were recruited from a community social and welfare association, the ‘Guangzhou Health and Happiness Association for Respectable Elders’ (GHHARE), which is unofficially aligned with the municipal government and has branches throughout all 10 districts of Guangzhou, and its memberships is open to Guangzhou local residents aged 50 years or older for a nominal monthly fee of four Renminbi (RMB) (one US dollar (USD) = seven RMB). The members of GHHARE covers about 7% of Guangzhou residents in this age group. Briefly, from September 2003 to January 2008, 30 430 from GHHARE were recruited. The baseline examination was conducted through face-to-face interview by trained nurses using a computer-assisted questionnaire in Guangzhou Twelfth People’s Hospital. The reliability and validity of the questionnaire were tested six months into recruitment by recalling 200 randomly selected participants for re-interview, and the results were satisfactory [20]. The GBCS was approved by the Guangzhou Medical Ethics Committee of the Chinese Medical Association and all participants gave written, informed consent before participation.

### Measurement of oral health

Oral health including frequency of toothbrushing, frequency of gum bleeding, and tooth loss was assessed using computer-assisted questionnaire. Frequency of toothbrushing was assessed by the question ‘How often do you brush your teeth?’ and the answers were categorised into ≥ twice/d, once/d, and < once/d. Tooth loss was assessed according to the number of missing teeth. Participants were asked ‘Do you have your own teeth?’ Those reported ‘No, dentures’ were categorised as edentulous, and others reported ‘Yes, but lost’ were further asked ‘How many teeth did you lose?’ to determine the number of missing teeth. To facilitate comparison to previous studies, the answers related to missing teeth were categorised into 0, 1–14, 15–28, >28, and edentulous [6,21,22]. In addition, participants were also asked ‘How often do your gums bleed when you brush?’ with four answers: ‘rarely’, ‘occasionally’, ‘sometimes’, and ‘always’. As gum bleeding occasionally might be caused by participants’ incorrect toothbrushing technique or vigorously hard brushing, rather than poor oral hygiene, we considered both gum bleeding occasionally or rarely as normal oral health and categorised frequency of gum bleeding into three groups: rarely/occasionally (normal), sometimes, and always.

### Mortality

Information on the underlying causes of deaths up to April 2021 was obtained via record linkage with the Death Registry of the Guangzhou Centre for Disease Control and Prevention (GZCDC). Causes of death were coded according to the 10th revisions of the International Classification of Diseases (ICD-10) by trained medical staff in each hospital. When the certificates were not issued by medical institutions (and hence might lead to a quality issue with the coding), the causes of death were verified by the GZCDC as part of its quality assurance programmed by cross-checking past medical history and conducting a verbal autopsy. The ICD-10 codes of the cause-specific mortality in this study were as follows: cardiovascular disease (CVD) (I00-I99, excluding I26, I27), ischemic heart disease (I20-I25), stroke (I60-I69), cancer (C00-C99) and respiratory disease (J00-J99). As numbers of events of site-specific cancer deaths were limited, only total cancer mortality but not site-specific cancer mortality analyses were included in the main analysis in our study.

### Potential confounders

Information of baseline demographic characteristics, socioeconomic status (SES) and lifestyle factors was asked using face-to-face interview by trained nurses and anthropometrics and physical examination were done. Demographic data including sex and age (years). Socioeconomic SES data was accounted for education level, household income, and occupation. Educational level was categorised into three groups: primary or below, middle school, and college and above. Household income was categorised into three groups: <30 000 Yuan/y, ≥30 000 Yuan/y, and unknown (one USD = seven Yuan). Occupation was classified into manual, non-manual, and others to reflect different types of job characteristics and associated socioeconomic implications. Status of smoking and alcohol use was assessed and classified as never, former, and current users. Physical activities were assessed using a validated Chinese version of International Physical Activity Questionnaire (IPAQ), and categorised into inactive, moderate and active [23]. Self-rated health was assessed and categorised into good and poor. Diabetes was defined as fasting glucose level ≥7.0 mmol per litre (mmol/L), self-reported physician-diagnosed diabetes, or current use of antidiabetic medications [20].

### Statistical analysis

Baseline characteristics were described using mean (standard deviation (SD)) for continuous variables and numbers (%) for categorical variables. One-way analysis of variance was used to compare differences for continuous variables and Pearson χ^2^ test for categorical variables. Kaplan-Meier survival curves were used to analyse associations of oral health status (i.e. toothbrushing frequency, gum bleeding, and number of missing teeth) with all-cause and cause-specific mortality. Cox regression was used to estimate hazard ratios (HRs) and the corresponding 95% confidence intervals (CIs) for the associations of oral health status with risks of all-cause and cause-specific mortality. Results were shown in forest plots. Potential confounders included sex, age, education level, occupation, household income, smoking status, alcohol use, physical activity, and self-rated health. In addition, diabetes was further adjusted in models exploring the association between number of missing teeth and mortality risk, and frequency of toothbrushing was further adjusted in models exploring the association between gum bleeding frequency and mortality risk.

Moreover, to explore potential effect modification, we also checked for interactions between oral health status and potential effect modifiers including age and education level on the risk of all-cause and cause-specific mortality. Stratification analyses were also done by age groups (<65 years and ≥65 years) and education level (primary or below and middle school and higher) to investigate potential variations in the associations of oral health status with all-cause and cause-specific mortality across different age groups. Additionally, to partly address reverse causality or confounding bias due to underlying illness, we conducted sensitivity analysis by excluding deaths occurring within the first three years of follow-up. Participants who died of any other causes were treated as censored at the date of death.

Furthermore, to assess if nutrition explained the associations of oral health with mortality, we calculated the percentage of excess risk mediated (PERM) for the following two nutritional factors: (1) total energy intake (kilocalorie per day, kcal/d) as continuous variable, those who reported unreliable dietary intake (total energy of <800 or >4200 kcal/d in men and of <600 or >3500 kcal/d in women) were excluded [24]; (2) dietary habit, which is categorised into five groups: vegetarians, special diet (for diabetes and renal disease), non-special diet, more meat, and more vegetables/fruits. For each nutritional factor, we estimated the PERM using the following formula:

PREM (%) = [(Hazard ratio (oral health + potential confounders)-hazard ratio (oral health + potential confounders + nutrition))/(Hazard ratio (oral health + potential confounders)-1)]*100%

All statistical analyses were conducted using Stata (version 16.0; STATA Crop LP, College Station, TX, USA). All tests were two-sided, and *P*-values <0.05 were considered as statistically significant.

## RESULTS

**Figure 1:**
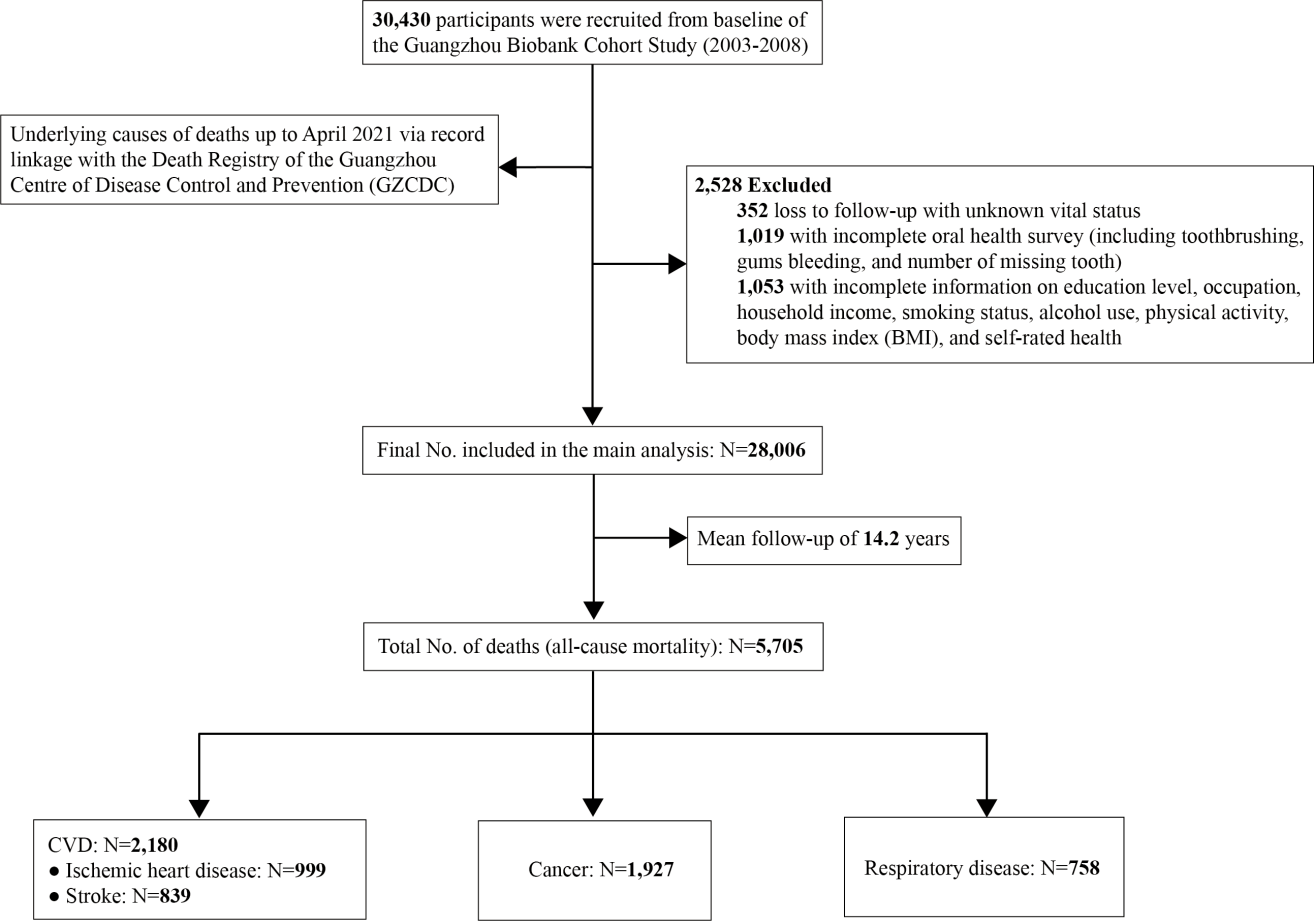
Flow diagram of study participants’ recruitment and main outcomes.

Of 30 430 participants at baseline, we excluded 352 who were lost to follow-up with unknown vital status, 1019 participants with missing data on oral health status and 1053 with missing data on covariates, yielding 28 006 participants (20 268 women and 7738 men) with all variables of interest in the current study. During an average of 14.2 years (SD = 3.2) with 399 128 person-years of follow-up, 5705 deaths were identified, including 2180 CVD (including 999 ischemic heart disease (IHD) and 839 stroke), 1927 cancer and 758 respiratory disease (**Figure 1**).

### Participants’ characteristics

**Table 1** shows that, of the 28 006 participants, the mean (SD) age was 62.0 (SD = 7.1) years, 20 268 (72.4%) were women, and the prevalence of toothbrushing of ≥ twice/d, once/d and < once/d was 69.6, 29.6, and 0.7%, respectively. Compared to those with toothbrushing ≥ twice/d, participants with toothbrushing once/d and < once/d had higher proportion of current or former smoking and alcohol use, but had lower SEP (i.e. lower education, manual occupation, and lower household income), proportion of physically active and good self-rated health. Furthermore, those with toothbrushing once/d and < once/d had more missing teeth and more who were edentulous.

**Table 1:** Baseline characteristics of 28 006 participants according to frequency of toothbrushing

Characteristics	Frequency of toothbrushing			All	*P*-value
	**≥twice/d**	**once/d**	**<once/d**		
Number of participants (%)	19 559 (69.6)	8331 (29.6)	195 (0.7)	28 006 (100.0)	-
Women, n (%)	14 710 (75.4)	5412 (65.2)	146 (74.9)	20 268 (72.4)	<0.001
Age in years, mean (SD)	61.5 (±7.0)	63.1 (±7.1)	65.6 (±7.1)	62.0 (±7.1)	0.83
Education level, n (%)					<0.001
*Primary or below*	7219 (37.0)	4697 (56.6)	116 (59.5)	12 032 (43.0)	
*Middle school*	10 276 (52.7)	3142 (37.8)	67 (34.4)	13 485 (48.1)	
*College or above*	2012 (10.3)	465 (5.6)	12 (6.1)	2489 (8.9)	
Occupation, n (%)					<0.001
*Manual*	8875 (45.5)	4883 (58.8)	132 (67.7)	13 890 (49.6)	
*Non-manual*	6758 (34.6)	2233 (26.8)	50 (25.6)	9031 (32.2)	
*Others*	3874 (19.9)	1198 (14.4)	13 (6.7)	5085 (18.2)	
Household income, Yuan/y (1 USD = 7 Yuan), n (%)					<0.001
*<30 000*	7062 (36.2)	3523 (42.4)	79 (40.5)	10 664 (38.1)	
*≥30 000*	8151 (41.8)	2487 (30.0)	41 (21.0)	10 679 (38.1)	
*Unknown*	4294 (22.0)	2294 (27.6)	195 (38.5)	6 663 (23.8)	
Smoking status, n (%)					<0.001
*Never*	16 394 (84.0)	6084 (73.3)	151 (77.4)	22 629 (80.8)	
*Former*	1541 (7.9)	1010 (12.1)	20 (10.3)	2571 (9.2)	
*Current*	1572 (8.1)	1210 (14.6)	24 (12.3)	2806 (10.0)	
Alcohol use, n (%)					<0.001
*Never*	14 260 (73.1)	5892 (71.0)	154 (79.0)	20 306 (72.5)	
*Former*	613 (3.1)	368 (4.4)	4 (2.0)	985 (3.5)	
*Current*	4634 (23.8)	2044 (24.6)	37 (19.0)	6715 (24.0)	
Physical activity, n (%)					<0.001
*Inactive*	1528 (7.8)	789 (9.5)	11 (5.6)	2328 (8.3)	
*Moderate*	7933 (40.7)	3612 (43.5)	78 (40.0)	11 623 (41.5)	
*Active*	10 046 (51.5)	3903 (47.0)	106 (54.4)	14 055 (50.2)	
Good self-rated health, n (%)	16 192 (83.0)	3315 (80.2)	172 (88.2)	23 020 (82.2)	<0.001
Frequency of gum bleeding					
*Rarely/occasionally*	17 642 (90.4)	7616 (91.7)	192 (98.5)	25 450 (90.9)	
*Sometimes*	1550 (8.0)	563 (6.8)	2 (1.0)	2115 (7.5)	
*Often*	315 (1.6)	125 (1.5)	1 (0.5)	441 (1.6)	
Number of missing teeth					<0.001
*0*	4952 (25.4)	1992 (24.0)	33 (16.9)	6977 (24.9)	
*1–14*	11 546 (59.2)	4515 (54.4)	103 (52.8)	16 164 (57.7)	
*15–28*	1784 (9.1)	1052 (12.7)	26 (13.3)	2862 (10.2)	
*>28*	231 (1.2)	120 (1.4)	5 (2.6)	356 (1.3)	
Edentulous	994 (5.1)	625 (7.5)	28 (14.4)	1647 (5.9)	

SD – standard deviation, USD – US dollar

Participants’ characteristics according to number of missing teeth and frequency of gum bleeding were shown in Tables S1–2 in the **Online Supplementary Document**.

### Association of oral health with all-cause and cause-specific mortality

The numbers of deaths and mortality rate for all-cause and cause-specific mortality by oral health groups were shown in Table S3 in the **Online Supplementary Document**. It shows that mortality rates for all-cause and cause-specific mortality increase with the reduction of toothbrushing and the increasing number of missing teeth.

**Figure 2:**
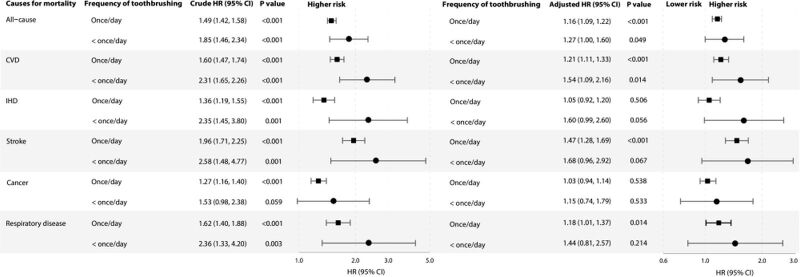
Associations of tooth brushing (vs.≥twice/d) with all-cause and cause-specific mortality in 28 006 participants of the Guangzhou Biobank Cohort Study recruited from September 2003 to January 2008 and followed up until April 2021. Forest plot showing HRs (log scale) and 95% CI (horisontal line). HRs were unadjusted in crude models, and were adjusted for sex, age, education level, occupation, household income, smoking status, alcohol use, physical activity, and self-rated health in multivariable-adjusted models. CVD – cardiovascular disease, IHD – ischemic heart disease, HR – hazard ratio, CI – confidence interval

**Figure 2** shows that, compared to those with toothbrushing frequency of ≥ twice/d, participants with lower toothbrushing frequency had higher risks of all-cause (HR = 1.16; 95% CI = 1.10, 1.22) for once/d and HR = 1.27, 95% CI = 1.00, 1.61 for < once/d; CVD (HR = 1.12; 95% CI = 1.12, 1.34) for once/d; and HR = 1.54, 95% CI = 1.09, 2.17 for < once/d; and stroke mortality (HR = 1.47; 95% CI = 1.28, 1.70) for once/d; and HR = 1.68; 95% CI = 0.96, 2.92 for < once/d) with dose-response patterns (all *P* for trend <0.001). Toothbrushing frequency of once/d and < once/d was also associated with higher risks of respiratory disease mortality (*P* for trend = 0.022), with adjusted HR = 1.18; 95% CI = 1.01, 1.37 and HR = 1.44; 95% CI = 0.81, 2.57, respectively. Significant association of toothbrushing of once/d and < once/d with IHD and cancer mortality was found in the crude models but attenuated and became non-significant in adjusted models (all *P* for trend >0.05).

**Figure 3:**
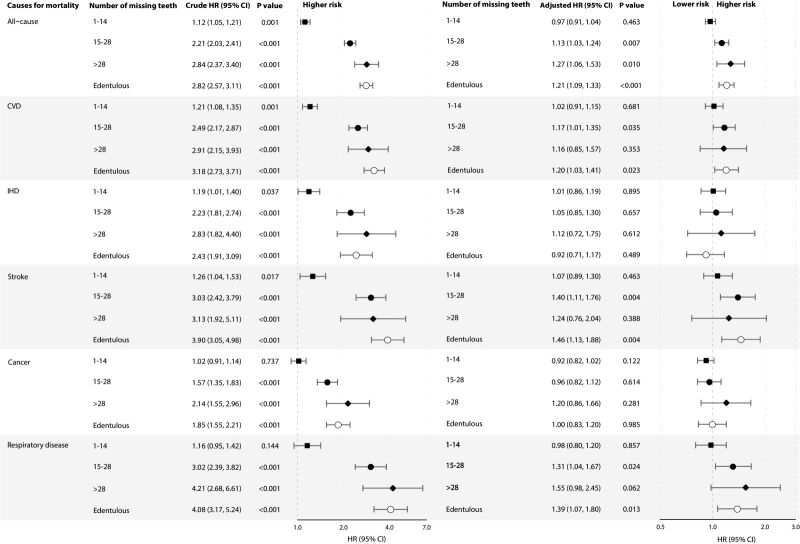
Associations of number of missing teeth (vs. 0) with all-cause and cause-specific mortality in 28 006 participants of the Guangzhou Biobank Cohort Study recruited from September 2003 to January 2008 and followed up until April 2021. Forest plot showing HRs (log scale) and 95% CI (horisontal line). HRs were unadjusted in crude models, and were adjusted for sex, age, education level, occupation, household income, smoking status, alcohol use, physical activity, self-rated health, and diabetes in multivariable-adjusted models. CVD – cardiovascular disease, IHD – ischemic heart disease, HR – hazard ratio, CI – confidence interval

**Figure 3** shows that, participants with greater number of missing teeth had higher risks of all-cause mortality with a dose-response pattern (*P* for trend <0.001), with adjusted HR = 1.13, 95% CI = 1.03, 1.24 for missing teeth of 15–28; HR = 1.27, 95% CI = 1.06, 1.51 for missing teeth of >28; and HR = 1.21, 95% CI = 1.09, 1.33 for who were edentulous, respectively. Similar dose-response patterns were also found for CVD, stroke, and respiratory disease mortality (all *P* for trend <0.01). Significant association of missing teeth with IHD and cancer mortality was found in the crude models but attenuated and became non-significant in adjusted models. Analyses of the associations of subgroups of toothbrushing frequency and number of missing teeth with all-cause and cause-specific mortality showed similar results (Figures S1–2 in the **Online Supplementary Document**). In addition, participants with frequent (always) gum bleeding had non-significantly higher risk of all-cause and CVD mortality, while those with less frequent (sometimes) gum bleeding had lower risks of all-cause, CVD, and IHD mortality (all *P* for trend <0.05). However, non-significant association of gum bleeding frequency with stroke, cancer nor respiratory disease mortality was found (all *P* for trend >0.05) (Figure S3 in the **Online Supplementary Document**).

### Sensitivity analysis

After excluding deaths within the first three years, the associations of toothbrushing frequency, number of missing teeth and gum bleeding frequency with all-cause and cause-specific mortality remained almost the same (Figures S6–8 in the **Online Supplementary Document**). Participants with missing teeth of 15–28 and who were edentulous also had significantly higher risks of all-cause, CVD, stroke and respiratory disease mortality with dose-response patterns (all *P* for trend <0.05), but not IHD and cancer mortality (all *P* for trend >0.05) (Figure S7 in the **Online Supplementary Document**). In addition, frequent (always) gum bleeding was non-significantly associated with higher risk of all-cause, CVD, and IHD mortality (all *P* for trend <0.05). Non-significant associations of frequency of gum bleeding frequency with stroke, cancer, nor respiratory disease mortality were found (Figure S8 in the **Online Supplementary Document**). After excluding those with poor self-rated health status, the associations of toothbrushing frequency, number of missing teeth and gum bleeding frequency with all-cause and cause-specific mortality remained (Tables S4–6 in the **Online Supplementary Document**). Participants with toothbrushing once/d (vs.≥twice/d) showed significantly higher risks of all-cause, CVD, and stroke mortality. However, the risks of respiratory disease attenuated and became non-significant (*P* for trend = 0.081) (Table S4 in the **Online Supplementary Document**). Participants with 15–28 missing teeth and who were edentulous also had significantly higher risks of all-cause and respiratory disease mortality with dose-response patterns (all *P* for trend <0.05). However, there was no similar increase in the risk of IHD and cancer mortality (all *P* for trend >0.05). Although the results for CVD and stroke mortality attenuated and became non-significant after multivariable adjustment, the dose response remained significant (all *P* for trend <0.05) (Table S5 in the **Online Supplementary Document**). In addition, frequent (always) gum bleeding was non-significantly associated with a higher risk of all-cause, CVD, and IHD mortality (all *P* for trend <0.05). Non-significant associations of frequency of gum bleeding frequency with stroke, cancer, nor respiratory disease mortality were found (Table S6 in the **Online Supplementary Document**). Although age interactions were not significant, age stratification analysis showed that, compared to toothbrushing at least twice/d, consistent and significant associations between less frequent toothbrushing of once/d and < once/d and mortality risks of all-cause, CVD, stroke and respiratory disease in participants aged ≥65 years. However, in those aged <65 years, the associations were non-significant. Non-significant association of toothbrushing frequency with cancer mortality was found in both subgroups (Figure S9 in the **Online Supplementary Document**). In addition, the association of missing teeth number with all-cause mortality was stronger in the older age group (*P* for interaction = 0.007). Participants who were edentulous or with missing teeth number of ≥15 had significantly higher risks of all-cause mortality in those aged ≥65 years, but not in the younger age group. No interactions between age and missing teeth number were found for cause-specific mortality (Figure S10 in the **Online Supplementary Document**). Associations of frequency of gum bleeding with all-cause, CVD, and cancer mortality varied by age (all *P* for interaction <0.05). They showed dose-response patterns, with lower risks observed only in the older group but not in the younger group (all *P* for trend <0.05). More frequent gum bleeding was also associated with lower risk of IHD mortality in older age group, showing a dose-response pattern (*P* for interaction = 0.226, *P* for trend = 0.005) (Figure S11 in the **Online Supplementary Document**). Additionally, while interactions with education were not significant (all *P* for interaction >0.05), stratified analysis by education level showed that a lower frequency of toothbrushing was associated with higher risks of all-cause, CVD, and stroke mortality in both lower-educated and higher-educated group with dose-response patterns (all *P* for trend <0.05). The dose-response pattern was stronger in the lower-educated group, and a significantly higher risk of IHD mortality with a dose-response pattern was observed only in the lower-educated group. Non-significant associations were found for cancer and respiratory disease mortality (Table S7 in the **Online Supplementary Document**). Moreover, associations of a greater number of missing teeth with all-cause, CVD, and stroke mortality were varied by education level (all *P* for interactions <0.05). Significant dose-response patterns and higher risks of all-cause, CVD, and stroke mortality were found in the lower-educated group, but not in the higher-educated group (Table S8 in the **Online Supplementary Document**). Furthermore, the association of frequency of gum bleeding with cancer mortality varied by education level (*P* for interaction = 0.049), but significant results was only found for dose-response in lower-educated groups (Table S9 in the **Online Supplementary Document**). Additionally, nutritional factors only weakly explained the associations of frequency of toothbrushing and number of missing teeth with all-cause, CVD, stroke, and respiratory disease mortality, with PERM% range 0.4–2.3% for total energy intake (Tables S10 and 12 in the **Online Supplementary Document**) and 0.0–1.6% for dietary habit (Tables S11 and S13 in the **Online Supplementary Document**). Non-significant results for IHD and cancer mortality were found.

## DISCUSSION

To our knowledge, our study is the largest-scale prospective cohort study investigating the association of specific toothbrushing frequency exposure with all-cause and cause-specific mortality and the dose-response patterns in older Chinese adults. In this prospective cohort study with 14 years of follow-up, less frequent toothbrushing and greater number of missing teeth were positively associated with all-cause mortality with a dose-response pattern, especially for CVD, stroke, and respiratory disease, but not associated with IHD and cancer mortality. In addition, education level modified the associations of missing teeth with risks of all-cause, CVD, and stroke mortality. The associations were stronger in lower-educated group.

Our findings were generally consistent with previous studies, indicating a consistent association between less frequent toothbrushing (toothbrushing of once/d and < once/d) with higher risks of all-cause and CVD mortality. The Chinese Kadoorie Biobank (CKB) study on 487 198 Chinese aged 30–79 years showed that, during 9.6 years of follow-up, participants who rarely or never brushed teeth, vs. those who brushed teeth regularly, had higher risks of all-cause death, major vascular events and stroke mortality, with HR = 1.25, 95% CI = 1.21, 1.28; HR = 1.12, 95% CI = 1.09, 1.15; and HR = 1.08, 95% CI = 1.05, 1.12, respectively [15]. However, the CKB study assessed toothbrushing behaviour using a simplified question about regular toothbrushing but did not specify toothbrushing frequency. An Iranian study of 50 045 adults aged 40–75 years indicated that toothbrushing once per day, vs. never brushing, was associated with approximately 20% lower risks of all-cause and CVD mortality. However, the association of toothbrushing of ≥ twice/d with all-cause and CVD mortality became non-significant after multivariate adjustment, which could be due to the small sample size [13]. Similar negative associations between toothbrushing and CVD risk was also found in three Korean studies [6,25,26] and a Scottish nationwide survey [27]. However, neither of the above investigated the dose-response patterns.

Our findings on respiratory mortality were also consistent with some previous studies. For example, the CKB showed that less frequent toothbrushing was associated with higher risks of chronic obstructive pulmonary disease (COPD) (HR = 1.12; 95% CI = 1.05, 1.20) [15]. In addition, two meta-analyses of randomised controlled trials showed that frequent toothbrushing and professional oral care were associated with lower risk of ventilator-associated pneumonia in critically ill patients, as well as inspiration pneumonia and mortality in older adults in aged care [28,29]. However, the participants included in the two meta-analyses were not community-based.

The associations of toothbrushing frequency with cancer risk reported in previous studies were controversial. One CKB study showed that compared to toothbrushing regularly, rarely or never brush teeth was associated with higher risk of cancer incidence and cancer mortality [15]. The other study showed that, compared to normal oral health (sometimes or rarely or never gum bleeding), rarely or never brush teeth was associated with a higher risk of total cancer mortality, while significant higher risks of site-specific cancer mortality were only specifically observed for oesophageal and liver cancer, but not others [8]. A meta-analysis including 28 case-control and two retrospective cohort studies showed that associations of higher toothbrushing frequency with lower gastric and upper aerodigestive cancer risk, but unified standard of toothbrushing frequency was not available [30].

Our results were consistent with a meta-analysis of 56 studies showing that edentulism was associated with higher risks of all-cause, CVD, and cerebrovascular diseases mortality [10]. A prospective cohort study on 36 283 Chinese elderly (median age = 90 years) also showed that participants with <20 natural teeth, vs. those with ≥20 natural teeth, was associated with higher risk of all-cause mortality (HR = 1.14; 95% CI = 1.06, 1.23) for those with 10–19 teeth; HR = 1.23, 95% CI = 1.15, 1.31 for those with 1–9 teeth; and HR = 1.35, 95% CI = 1.26, 1.44 for those without natural teeth) [17]. Consistent results were observed in most previous studies from different settings [31–33], although a few studies showed inconsistent results. One Finnish case-control cohort study including coronary artery disease patients and healthy controls showed that an increasing number of teeth (per ten teeth), vs. being edentulous, was not associated with a lower risk of all-cause mortality [34]. Another study on 57 001 postmenopausal women found non-significant association of edentulism with CVD mortality after multivariate adjustment [35]. Furthermore, the other study on 50 884 women showed non-significant association of tooth loss (vs. those without tooth loss) with stroke mortality [36]. The discrepancies could be due to variations in the oral health measurement instruments used across studies. Specifically, some studies assessed numbers of missing teeth [17,32,34], albeit with minor differences in categorical classification, while other studies used a simplified question regarding the presence of periodontitis or edentulism among participants [35,36]. As reported in a meta-analysis, total tooth loss in adult patients was a recognised consequence of severe periodontitis. The observed elevated estimates of associations between greater numbers of missing teeth, as well as edentulism, and all-cause/cause-specific mortality may be indictive of a potential dose-dependent relationship [10]. By incorporating both the assessment of missing teeth and edentulism, our study contributes to a more comprehensive understanding of the relationship between tooth loss and health outcomes, thereby offering more robust evidence. Furthermore, variations in participant selection criteria have contributed to limitation of the observed associations. Specifically, the inclusion of solely female participants [35,36] and the incorporation of clinical patients [34] may limit the generalisability of the results. In addition, smaller sample sizes and case counts represent significant concern. Compared to other large studies, the Finnish study included a limited sample size of 473 individuals [34]. Therefore, these factors may lead to potential underestimation of the associations.

The evidence regarding association of gum bleeding with mortality risk was limited. The CKB study showed similar results. Among those who brushed teeth regularly, less frequent (sometimes) gum bleeding but not more frequent (always) gum bleeding was associated with lower risks of all-cause, major vascular events, major coronary events, and IHD mortality, whereas both non-significant results were observed for stroke and COPD mortality [15].

In our analysis, we observed that lower levels of education were associated with poorer oral health outcomes. This observation prompts further exploration into how broader socioeconomic factors influence oral health. Socioeconomic status, including income, education, and occupational class, has been shown to affect health behaviours, including oral hygiene practices and utilisation of dental care services. Low SES often correlates with reduced access to health resources, including preventive dental care, which can lead to a higher prevalence of oral health issues [37–41]. Furthermore, socioeconomic disparities can influence the affordability and prioritisation of oral health care, potentially leading to delayed treatment and poorer health outcomes. Such disparities may also influence dietary choices and other health behaviours related to oral conditions, thereby exacerbating the risk of associated with morbidities and mortality. By understanding these dynamics, interventions can be better tailored to address the specific needs of lower SES groups, potentially reducing disparities in oral health and improving overall health outcomes.

Anatomically, the oral cavity serves as a vital interface facilitating exchanges between the internal and external environments of human beings. A 2021 review has outlined the mechanisms that link periodontitis to extra-oral comorbidities, which are consistent with clinical observations that associated periodontitis with bacteraemia, low-grade systemic inflammation, increased myelopoietic activity and the effectiveness of localised periodontal treatment to mitigate systemic inflammatory markers and enhance the activity of comorbid diseases (as indicated by surrogate markers) [42]. Oral hygiene alters the oral microbiota. Regular toothbrushing and periodontal treatment has been reported to be associated with lower levels of inflammatory markers such as C-reactive protein and interleukins [27]. In contrast, poor oral hygiene facilitates the growth of microbial oral biofilms, leading to an increased presence of free microorganisms in the saliva [43]. Dysbiosis of the oral microbiota is a feature of periodontal diseases’ initiation and progression [44,45], which has increasingly been linked to CVD [42]. The abundance and diversity of oral microbiome are second only to the gut microbiome, harbouring over 700 species of bacteria [46]. Periodontal disease originates in response to a specific group of periodopathogens [47,48]; predominantly, Gram-negative anaerobes, such as *P. gingivalis*, *F. nucleatum*, *A. actinomycetemcomitans*, *T. forsythia*, *T. enticola* and *T. spirochetes*, which increase in the subgingival biofilm during the disease. Bacteria infiltrate tissues and create periodontal pockets that represent a microenvironment where the balance between microbes and immune response is disrupted through direct and indirect mechanisms [48,49]. The related inflammatory response is characterised by the local production of various pro-inflammatory mediators, including C-reactive protein, interleukin-1β, interleukin −6, tumour necrosis factor-α, matrix metalloproteinases, and interferon-gamma [50]. As a result of the increase in these mediators, the degree of destruction of periodontal tissues is accelerated. Compared to healthy controls, patients with severe periodontitis and elevated levels of proinflammatory mediators, such as interleukin-1, interleukin-6, C-reactive protein, and fibrinogen, also had increased neutrophils count in the peripheral blood [51,52]. Many diseases associated with oral health disturbances, including CVDs, show alterations in the composition of the blood microbiota and circulating neutrophils phenotypes [52]. Endotoxin from oral microbes may vary depending on bacterial communities and oral health status, thereby differentially affecting cellular lipopolysaccharide tolerance mechanisms that contribute to the neutrophil phenotype typical of periodontitis [52]. In parallel, neutrophil extracellular traps were found to contribute to atherosclerosis and thrombosis [52]. Furthermore, tooth loss can be an outcome of chronic periodontal disease, whereby bacteria originating from periodontal lesions may be released into the circulation. This hematogenous dissemination of the periodontal bacteria or the release of inflammatory mediators from periodontal tissues into the bloodstream is likely to trigger systemic inflammation [42]. Likewise, the inflammatory immune response may contribute to the underlying mechanism linking oral health to atherosclerotic and CVD risk. This can occur through the release of inflammatory mediators from the periodontium into the bloodstream, or via systemically induced inflammatory mechanisms coupled with platelet activation or dyslipidaemia [53]. Moreover, individuals with late-life illnesses [54] and physical frailty [55] might have a greater number of missing teeth, which in turn might influence their lifestyle. For example, they might brush their teeth less frequently. However, our study attempted to minimise this bias through comprehensive adjustment for a wide range of confounding factors and showed results mostly consistent results with previous studies.

The strengths of our study included the prospective cohort study design, large sample size, long follow-up with large numbers of death, and assessment of mortality outcomes. However, there were some limitations. First, in the current study, oral health was assessed only by interview. As oral health was assessed by self-reporting, reporting error was possible. However, information on toothbrushing and missing teeth can generally be reliably reported by most adults, although factors such as gum bleeding might not be easily quantifiable. We also did not have comparable data prior to baseline and during follow-up. Objective dental examinations, such as radiographic panoramic examination and periodontal probing by dentists were not feasible for such a large cohort. The absence of objective measures might lead to underestimation of the strengths of the associations. Second, our study did not include information on factors such as dental visit history, periodontal disease, dental cleaning, dental caries, masticatory function, and oral microbiota, which were associated with CVD and respiratory risk in previous studies [6,10,36]. However, as the accessibility and utilisation of dental services in our study population were generally low, the absence of this information might not have a substantial impact on the results. Further studies incorporating a broader range of oral health indicators, including professional assessments and detailed hygiene practices, to provide a more detailed understanding of the association between oral health and overall health outcomes are warranted. Third, reverse causality was another potential concern. However, sensitivity analysis excluding deaths occurring within the first three years showed similar results. Fourth, we did not have detailed data on the specific causes of tooth loss, which may include factors related to aging such as gum disease and bone loss. These factors themselves could be linked to systematic health and hence to mortality, potentially confounding our results. Aging might not be only leading to natural tooth loss and reduced oral hygiene, but could also be associated with an increased overall risk of mortality. To mitigate the potential impact of reverse causality, we conducted a sensitivity analysis by excluding deaths that occurred within the first three years of follow-up. The results of this analysis did not significantly differ from our main findings, suggesting that the observed associations were not solely due to immediate pre-existing health conditions leading to death. Nonetheless, the possibility of underlying senescence affecting both the exposure and the outcomes cannot be completely ruled out. Fifth, as oral health information was only collected at baseline in GBCS, we were unable to assess longitudinal changes in oral health with their impact on mortality over time. However, it is important to note that frequency of toothbrushing may be a long-term habit that does not significantly change over time. Future studies investigating the association of changes of missing teeth with mortality to provide deeper insights into their dynamics are needed. Finally, cultural and regional specificity might be another limitation. As all GBCS participants were older Chinese adults, the generalisability of our findings to younger age, other demographics, or Western populations may be limited.

## CONCLUSIONS

In summary, in older Chinese people, lower frequency of toothbrushing and a greater number of missing teeth were both associated with higher risks of all-cause mortality with dose-response patterns, especially for CVD, stroke, and respiratory disease mortality. The dose-response patterns were stronger in lower-educated participants. These findings suggest that public health messages to encourage good oral hygiene and implementing initiatives of comprehensive oral hygiene promotion campaigns, such as regular tooth brushing, and raising awareness of and addressing tooth loss, especially for lower-educated residents, would be valuable to attenuate the risk of mortality in older adults.

## Additional material


Online Supplementary Document
