# Supplementary material for: Association of oral health with all-cause and cause-specific mortality in older Chinese adults: A 14-year follow-up of the Guangzhou Biobank Cohort study
Source: J Glob Health. 2024 Jul 5;14:04111. doi: 10.7189/jogh.14.04111 (PMC11225964; doi:10.7189/jogh.14.04111)
Supplement: Online Supplementary Document [file jogh-14-04111-s001.pdf]

Association of oral health with all-cause and cause-specific mortality in older Chinese: a 14-year prospective cohort study of the Guangzhou Biobank Cohort study

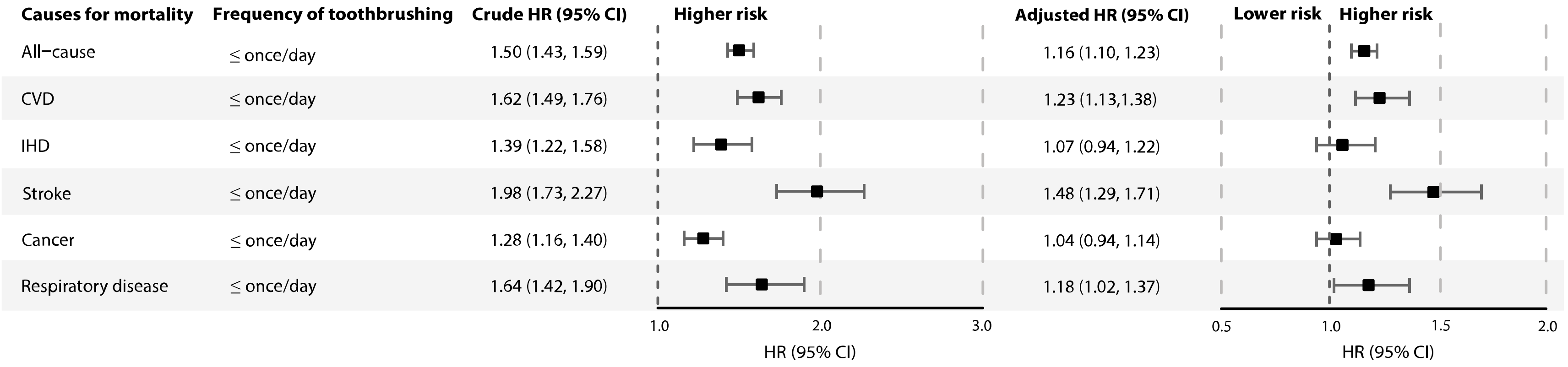

Supplementary Figure 1. Associations of frequency of toothbrushing with 2 subgroups (≤once/day versus ≥twice/day) with all-cause and cause-specific mortality on 28,006 participants of the Guangzhou Biobank Cohort Study recruited from September 2003 to January 2008 and followed up until April 2021. Forest plot showing HRs in circles and 95% CI (horizontal line). HRs were unadjusted in crude models, and were adjusted for sex, age, education level, occupation, household income, smoking status, alcohol use, physical activity, and self-rated health in multivariable-adjusted models.

CVD = cardiovascular disease, IHD = ischemic heart disease, HR = hazard ratio, CI = confidence interval.

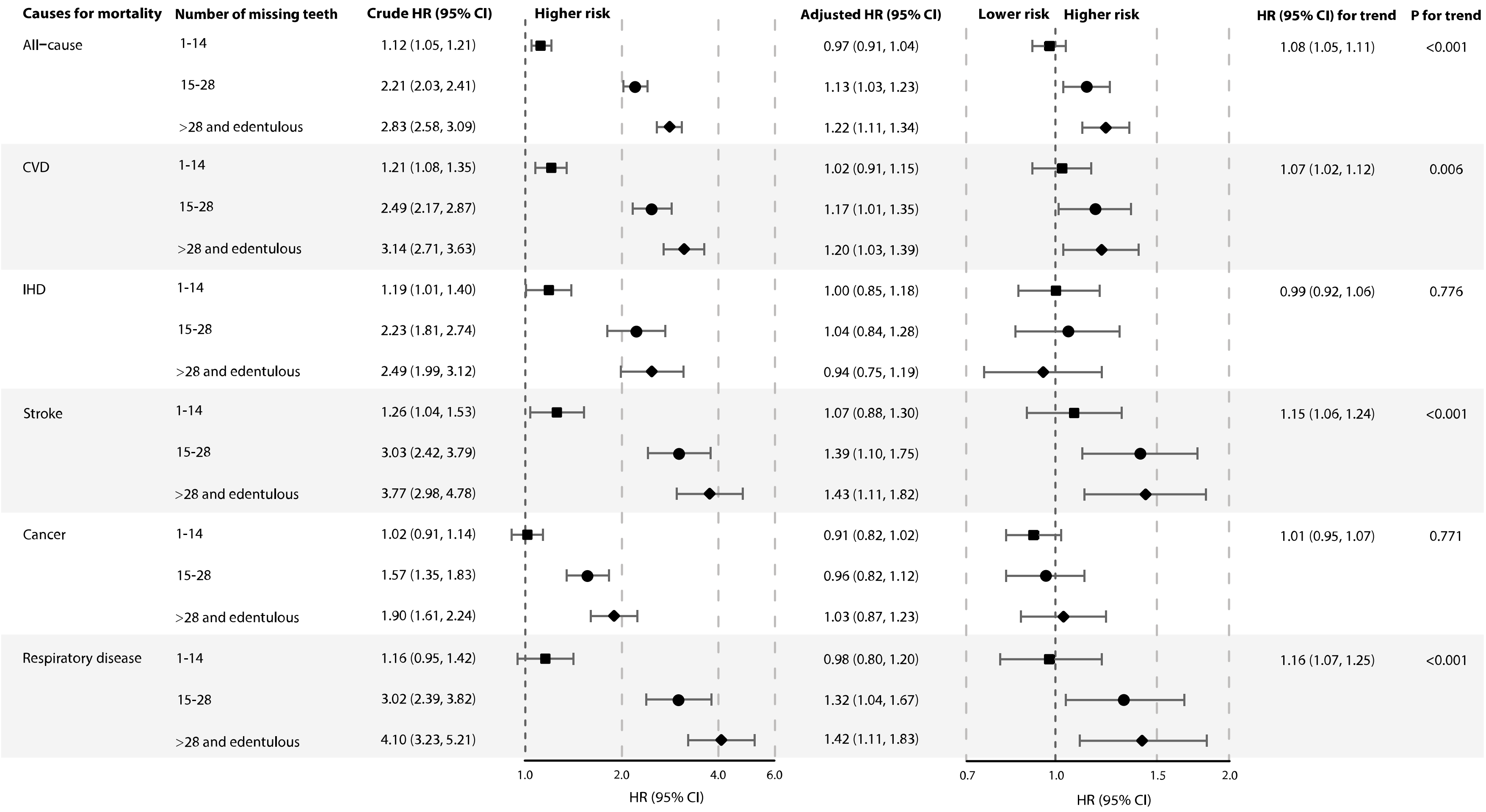

Supplementary Figure 2. Associations of number of missing teeth with 4 subgroups (3 subgroups versus 0) with all-cause and cause-specific mortality in 28,006 participants

of the Guangzhou Biobank Cohort Study recruited from September 2003 to January 2008 and followed up until April 2021. Forest plot showing HRs (log scale) in circles and 95% CI (horizontal line). HRs were unadjusted in crude models, and were adjusting for sex, age, education level, occupation, household income, smoking status, alcohol use, physical activity, self-rated health, and diabetes in multivariable-adjusted models.

CVD = cardiovascular disease, IHD = ischemic heart disease, HR = hazard ratio, CI = confidence interval.

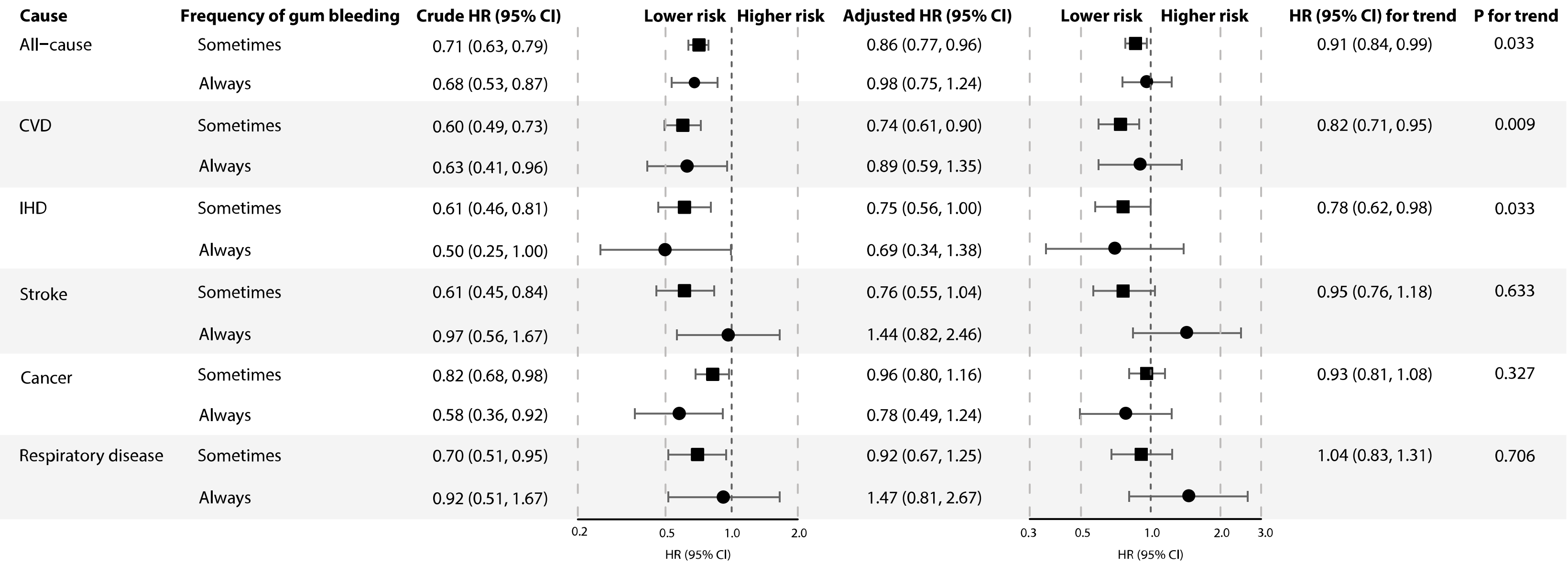

Supplementary Figure 3. Associations of gum bleeding (versus occasionally/rarely) with all-cause and cause-specific mortality in 28,006 participants of the Guangzhou Biobank Cohort Study recruited from September 2003 to January 2008 and followed up until April 2021. Forest plot showing HRs (log scale) and 95% CI (horizontal line). HRs were unadjusted in crude models, and were adjusted for sex, age, education level, occupation, household income, smoking status, alcohol use, physical activity, self-rated health, and frequency of toothbrushing in multivariable-adjusted models.

CVD = cardiovascular disease, IHD = ischemic heart disease, HR = hazard ratio, CI = confidence interval.

Supplementary Table 1. Baseline characteristics of 28,006 participants according to number of missing teeth

Characteristics	Number of missing teeth					All	P value
	0	1-14	15-28	>28	Edentulous		
Number (%)	6,977 (24.9)	16,164 (57.7)	2,862 (10.2)	356 (1.3)	1,647 (5.9)	28,006 (100.0)	-
Women, n (%)	5,114 (73.3)	11,757 (72.7)	1,987 (69.4)	246 (69.1)	1,164 (70.7)	20,268 (72.4)	<0.001
Age, years, mean (SD)	59.7 (6.5)	61.4 (6.7)	66.6 (6.4)	67.9 (6.5)	68.6 (6.4)	62.0 (7.1)	0.001
Education level, n (%)							<0.001
Primary or below	2,423 (34.7)	6,541 (40.5)	1,729 (60.4)	243 (68.3)	1,096 (66.5)	12,032 (43.0)	
Middle school	3,878 (55.6)	8,113 (50.2)	941 (32.9)	97 (27.2)	456 (27.7)	13,485 (48.1)	
College	676 (9.7)	1,510 (9.3)	192 (6.7)	16 (4.5)	95 (5.8)	2,489 (8.9)	
Occupation, n (%)							<0.001
Manual	3,390 (48.6)	7,675 (47.5)	1,625 (56.8)	194 (54.5)	1,006 (61.1)	13,890 (49.6)	
Non-manual	2,336 (33.5)	5,326 (32.9)	831 (29.0)	95 (26.7)	443 (26.9)	9,031 (32.2)	

Others	1,251 (17.9)	3,163 (19.6)	406 (14.2)	67 (18.8)	198 (12.0)	5,085 (18.2)	
Household income, RMB/year, n (%)							<0.001
<30,000	2,564 (36.7)	5,844 (36.2)	1,278 (44.6)	173 (48.6)	805 (48.9)	10,664 (38.1)	
≥30,000	2,873 (41.2)	6,647 (41.1)	732 (25.6)	82 (23.0)	345 (20.9)	10,679 (38.1)	
Unknown	1,540 (22.1)	3,673 (22.7)	852 (29.8)	101 (28.4)	497 (30.2)	6,663 (23.8)	
Smoking status, n (%)							<0.001
Never	5,818 (83.4)	13,256 (82.0)	2,133 (74.5)	244 (68.5)	1,178 (71.5)	22,629 (80.8)	
Former	551 (7.9)	1,378 (8.5)	360 (12.6)	49 (13.8)	233 (14.2)	2,571 (9.2)	
Current	608 (8.7)	1,530 (9.5)	369 (12.9)	63 (17.7)	236 (14.3)	2,806 (10.0)	
Alcohol use, n (%)							<0.001
Never	5,070 (72.7)	11,587 (71.7)	2,128 (74.3)	256 (71.9)	1,265 (76.8)	20,306 (72.5)	
Former	228 (3.3)	578 (3.6)	107 (3.7)	15 (4.2)	57 (3.5)	985 (3.5)	
Current	1,679 (24.0)	3,999 (24.7)	627 (21.9)	85 (23.9)	325 (19.7)	6,715 (24.0)	

Physical activity, n (%)							<0.001
Inactive	588 (8.4)	1,391 (8.6)	210 (7.4)	38 (10.7)	101 (6.1)	2,328 (8.3)	
Moderate	2,816 (40.4)	6,724 (41.6)	1,217 (42.5)	170 (47.7)	696 (42.3)	11,623 (41.5)	
Active	3,573 (51.2)	8,049 (49.8)	1,435 (50.1)	148 (41.6)	850 (51.6)	14,055 (50.2)	
Good self-rated health, n (%)	5,853 (83.9)	13,313 (82.4)	2,269 (79.3)	273 (76.7)	1,312 (79.7)	23.020 (82.2)	<0.0011
Frequency of toothbrushing							<0.001
≥twice/day	4,952 (71.0)	11,546 (71.5)	1,784 (62.3)	231 (64.9)	994 (60.3)	19,507 (69.7)	
Once/day	1,992 (28.5)	4,515 (27.9)	1,052 (36.8)	120 (33.7)	625 (38.0)	8,304 (29.6)	
<once/day	33 (0.5)	103 (0.6)	26 (0.9)	5 (1.4)	28 (1.7)	195 (0.7)	
Frequency of gum bleeding							<0.001
Rarely/occasionally	6,356 (91.1)	14,595 (90.3)	2,576 (90.0)	341 (95.8)	1,582 (96.1)	25,450 (90.9)	
Sometimes	526 (7.5)	1,287 (8.0)	243 (8.5)	14 (3.9)	45 (2.7)	2,115 (7.5)	
Always	95 (1.4)	282 (1.7)	43 (1.5)	1 (0.3)	20 (1.2)	441 (1.6)	

Diabetes					
No	6,121 (87.7)	14,035 (86.8)	2,379 (83.1)	303 (85.1)	1,366 (82.9)
Yes	856 (12.3)	2,129 (13.2)	483 (16.9)	53 (14.9)	281 (17.1)

SD = standard deviation.

Supplementary Table 2. Baseline characteristics of 28,006 participants according to frequency of gum bleeding

Characteristics	Frequency of gum bleeding			All	P value
	Rarely/occasionally	Sometimes	Always		
Number (%)	25,450 (90.9)	2,115 (7.5)	441 (1.6)	28,006 (100.0)	-
Women, n (%)	18,304 (71.9)	1,609 (76.1)	355 (80.5)	20,268 (72.4)	<0.001
Age, years, mean (SD)	62.2 (7.1)	60.6 (6.6)	59.4 (6.4)	62.0 (7.1)	<0.001
Education level, n (%)					0.004

Primary or below	11,005 (43.2)	836 (39.5)	191 (43.3)	12,032 (43.0)	
Middle school	12,187 (47.9)	1,075 (50.8)	223 (50.6)	13,485 (48.1)	
College	2,258 (8.9)	204 (9.7)	27 (6.1)	2,489 (8.9)	
Occupation, n (%)					0.177
Manual	12,616 (49.6)	1,045 (49.4)	229 (51.9)	13,890 (49.6)	
Non-manual	8,246 (32.4)	661 (31.3)	124 (28.1)	9,031 (32.2)	
Others	4,588 (18.0)	409 (19.3)	88 (20.0)	5,085 (18.2)	
Household income, RMB/year, n (%)					0.041
<30,000	9,741 (38.3)	767 (36.3)	156 (35.4)	10,664 (38.1)	
≥30,000	9,646 (37.9)	840 (39.7)	193 (43.7)	10,679 (38.1)	
Unknown	6,063 (23.8)	508 (24.0)	92 (20.9)	6,663 (23.8)	
Smoking status, n (%)					<0.001
Never	20,414 (80.2)	1,819 (86.0)	396 (89.8)	22,629 (80.8)	

Former	2,377 (9.3)	166 (7.9)	28 (6.3)	2,571 (9.2)	
Current	2,659 (10.5)	130 (6.1)	17 (3.9)	2,806 (10.0)	
Alcohol use, n (%)					0.167
Never	18,400 (72.3)	1,580 (74.7)	326 (73.9)	20,306 (72.5)	
Former	897 (3.5)	72 (3.4)	16 (3.6)	985 (3.5)	
Current	6,153 (24.2)	463 (21.9)	99 (22.5)	6,715 (24.0)	
Physical activity, n (%)					0.001
Inactive	2,064 (8.1)	219 (10.3)	45 (10.2)	2,328 (8.3)	
Moderate	10,545 (41.4)	881 (41.7)	197 (44.7)	11,623 (41.5)	
Active	12,841 (50.5)	1,015 (48.0)	199 (45.1)	14,055 (50.2)	
Good self-rated health, n (%)	21,023 (82.6)	1,675 (79.2)	322 (73.0)	23.020 (82.2)	<0.001
Frequency of toothbrushing					<0.001
≥twice/day	17,642 (69.3)	1,550 (73.3)	315 (71.4)	19,507 (69.7)	

Once/day	7,616 (29.9)	563 (26.6)	125 (28.3)	8,304 (29.6)	
<once/day	192 (0.8)	2 (0.1)	1 (0.2)	195 (0.7)	
Number of missing teeth					<0.001
0	6,356 (25.0)	526 (24.9)	95 (21.5)	6,977 (24.9)	
1-14	14,595 (57.4)	1,287 (60.8)	282 (64.0)	16,164 (57.7)	
15-28	2,576 (10.1)	243 (11.5)	43 (9.8)	2,862 (10.2)	
>28	341 (1.3)	14 (0.7)	1 (0.2)	356 (1.3)	
Edentulous	1,582 (6.2)	45 (2.1)	20 (4.5)	1,647 (5.9)	

SD = standard deviation.

Supplementary Table 3. Number of deaths and mortality rate for all-cause and cause-specific mortality by frequency of toothbrushing and number of missing teeth on 28,006 participants of the Guangzhou Biobank Cohort Study recruited from September 2003 to January 2008 and followed up until April 2021

	**No. of deaths/ person-years**	**Mortality rate, per 10,000 person-years**	**No. of deaths/ person-years**	**Mortality rate, per 10,000 person-years**	**No. of deaths/ person-years**	**Mortality rate, per 10,000 person-years**
	**All-cause mortality**		**CVD mortality**		**IHD mortality**	
**Frequency of toothbrushing**						
≥twice/day	3,485/280,218	124.37	1,293/280,218	46.14	629/280,218	22.45
Once/day	2,149/116,066	185.15	853/116,066	73.49	353/116,066	30.41
<once/day	71/2,844	249.66	34/2,844	119.56	17/2,844	59.78
**Number of missing teeth**						
0	1,132/101,812	111.18	406/101,812	39.88	197/101,812	19.35
1-14	2,835/231,773	122.32	1,081/231,773	46.64	517/231,773	22.31

15-28	936/39,135	239.17	376/39,135	96.08	163/39,135	41.65
>28	131/4,523	289.62	47/4,523	103.91	22/4,523	48.64
Edentulous	671/21,884	306.62	270/21,884	123.38	100/21,884	45.70
	**Stroke mortality**		**Cancer mortality**		**Respiratory disease mortality**	
**Frequency of toothbrushing**						
≥twice/day	457/280,218	16.31	1,251/280,218	44.64	447/280,218	15.95
Once/day	369/116,066	31.79	656/116,066	56.52	299/116,066	25.76
<once/day	13/2,844	45.71	20/2,844	70.32	12/2,844	42.20
**Number of missing teeth**						
0	143/101,812	14.05	441/101,812	43.31	406/101,812	13.06
1-14	401/231,773	17.30	1,013/231,773	43.71	1,081/231,773	14.71
15-28	161/39,135	41.14	261/39,135	66.69	376/39,135	38.07
>28	18/4,523	39.79	40/4,523	88.43	47/4,523	48.64

Edentulous	116/21,884	53.01	172/21,884	78.60	270/21,884	51.64

CVD = cardiovascular disease, IHD = ischemic heart disease.

All-cause mortality

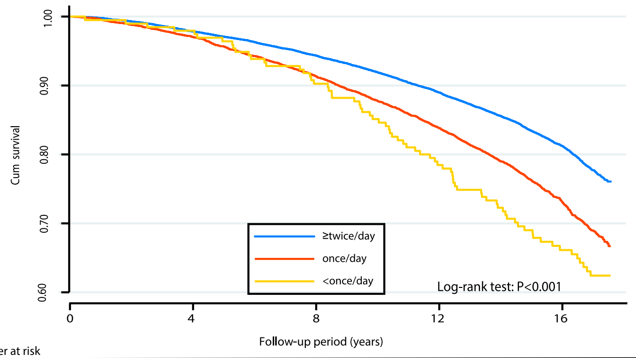

Number at risk

≥twice/day	19,507	19,086	18,346	17,302	4,925
once/day	8,304	8,055	7,562	6,935	2,183
<once/day	195	191	176	153	108

Total CVD mortality

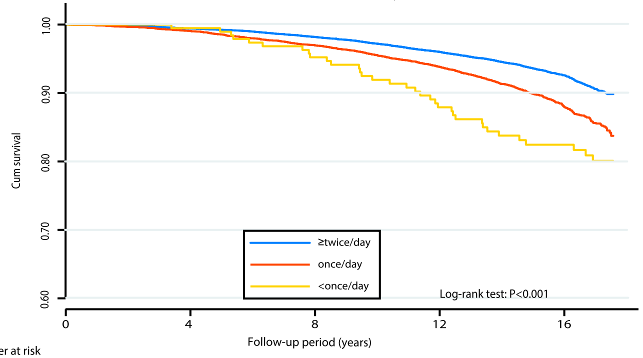

Number at risk

≥twice/day	19,507	19,086	18,346	17,302	4,925
once/day	8,304	8,055	7,562	6,935	2,183
<once/day	195	191	176	153	108

IHD mortality

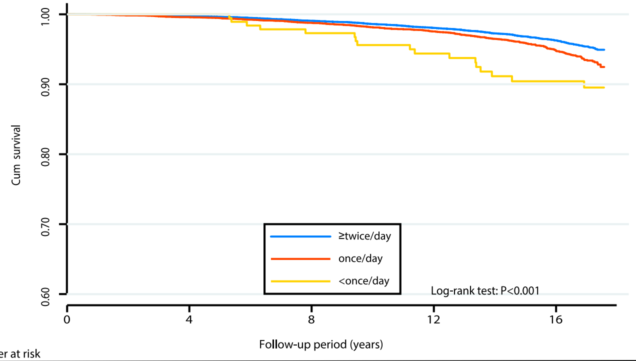

Number at risk

≥twice/day	19,507	19,086	18,346	17,302	4,925
once/day	8,304	8,055	7,562	6,935	2,183
<once/day	195	191	176	153	108

Total stroke mortality

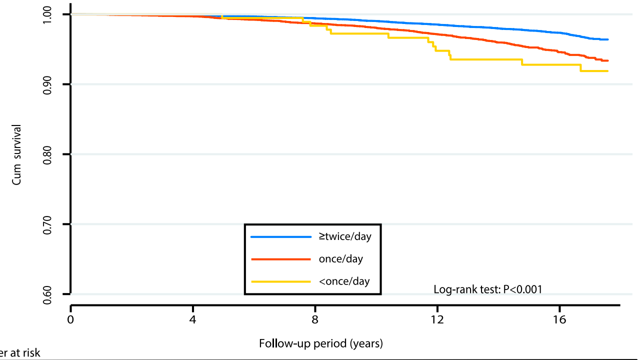

Number at risk

≥twice/day	19,507	19,086	18,346	17,302	4,925
once/day	8,304	8,055	7,562	6,935	2,183
<once/day	195	191	176	153	108

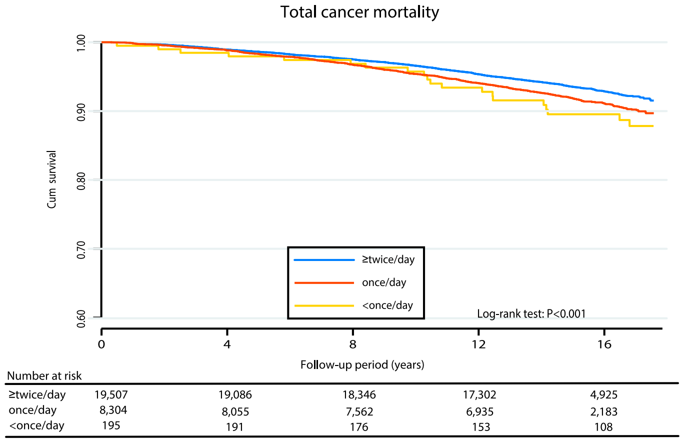

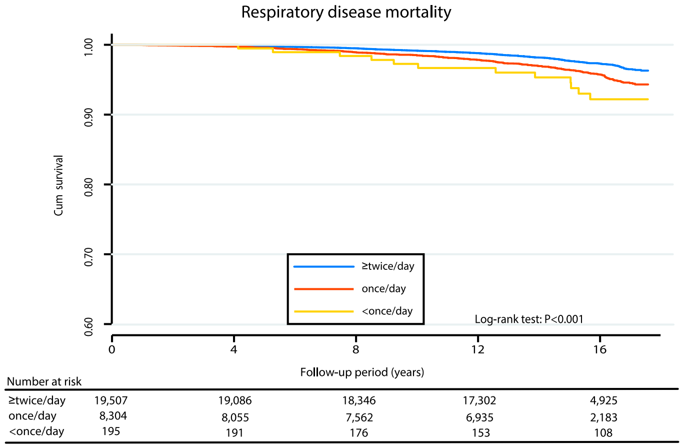

Supplementary Figure 4. Kaplan-Meier analyses for association of frequency of toothbrushing (≥twice/day, once/day, and <once/day) with all-cause and cause-specific mortality. CVD = cardiovascular disease, IHD = ischemic heart disease.

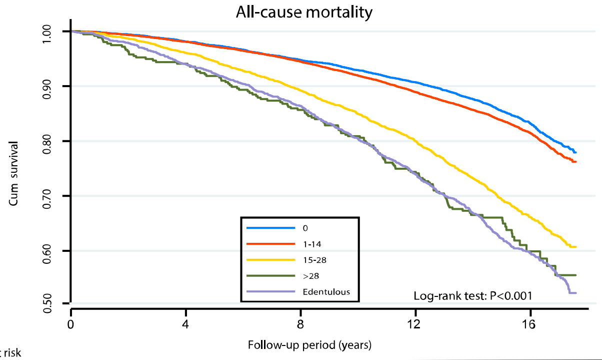

Number at risk					
0	6,977	6,844	6,587	6,302	2,093
1-14	16,164	15,856	15,225	14,327	3,776
15-28	2,862	2,749	2,547	2,283	794
>28	356	335	304	264	46
Edentulous	1,647	1,548	1,421	1,214	507

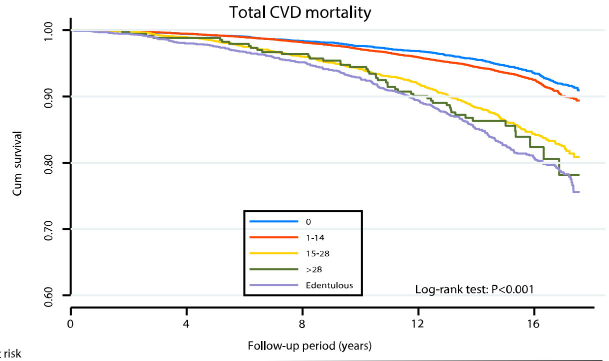

Number at risk					
0	6,977	6,844	6,587	6,302	2,093
1-14	16,164	15,856	15,225	14,327	3,776
15-28	2,862	2,749	2,547	2,283	794
>28	356	335	304	264	46
Edentulous	1,647	1,548	1,421	1,214	507

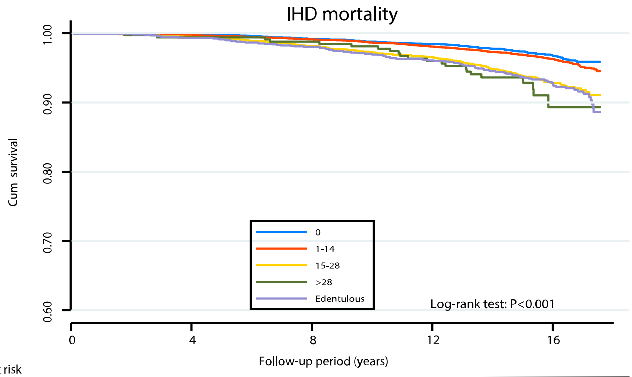

Number at risk					
0	6,977	6,844	6,587	6,302	2,093
1-14	16,164	15,856	15,225	14,327	3,776
15-28	2,862	2,749	2,547	2,283	794
>28	356	335	304	264	46
Edentulous	1,647	1,548	1,421	1,214	507

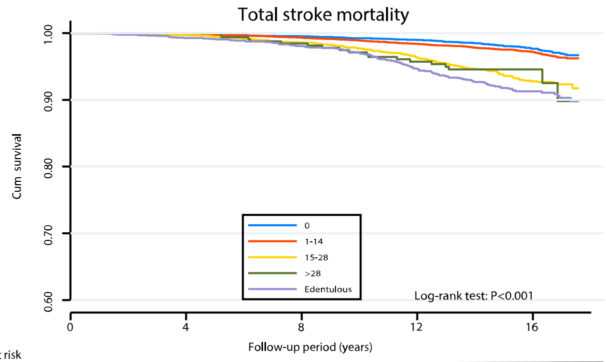

Number at risk					
0	6,977	6,844	6,587	6,302	2,093
1-14	16,164	15,856	15,225	14,327	3,776
15-28	2,862	2,749	2,547	2,283	794
>28	356	335	304	264	46
Edentulous	1,647	1,548	1,421	1,214	507

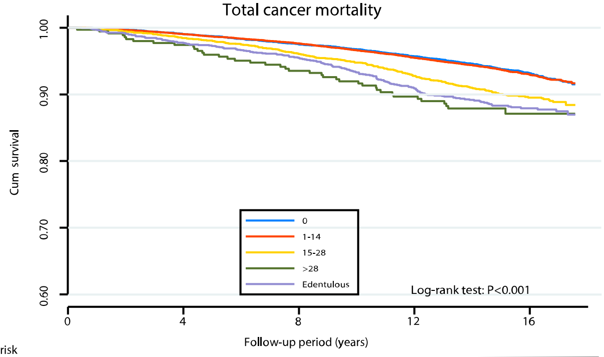

Number at risk					
0	6,977	6,844	6,587	6,302	2,093
1-14	16,164	15,856	15,225	14,327	3,776
15-28	2,862	2,749	2,547	2,283	794
>28	356	335	304	264	46
Edentulous	1,647	1,548	1,421	1,214	507

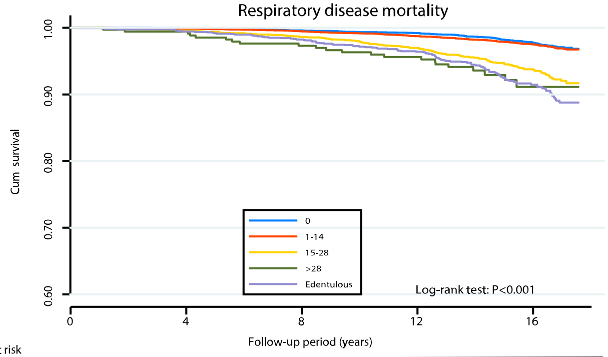

Number at risk					
0	6,977	6,844	6,587	6,302	2,093
1-14	16,164	15,856	15,225	14,327	3,776
15-28	2,862	2,749	2,547	2,283	794
>28	356	335	304	264	46
Edentulous	1,647	1,548	1,421	1,214	507

Supplementary Figure 5. Kaplan-Meier analyses for association of number of missing teeth (0, 1-14, 15-28, >28, edentulous) with all-cause and cause-specific mortality.

CVD = cardiovascular disease, IHD = ischemic heart disease.

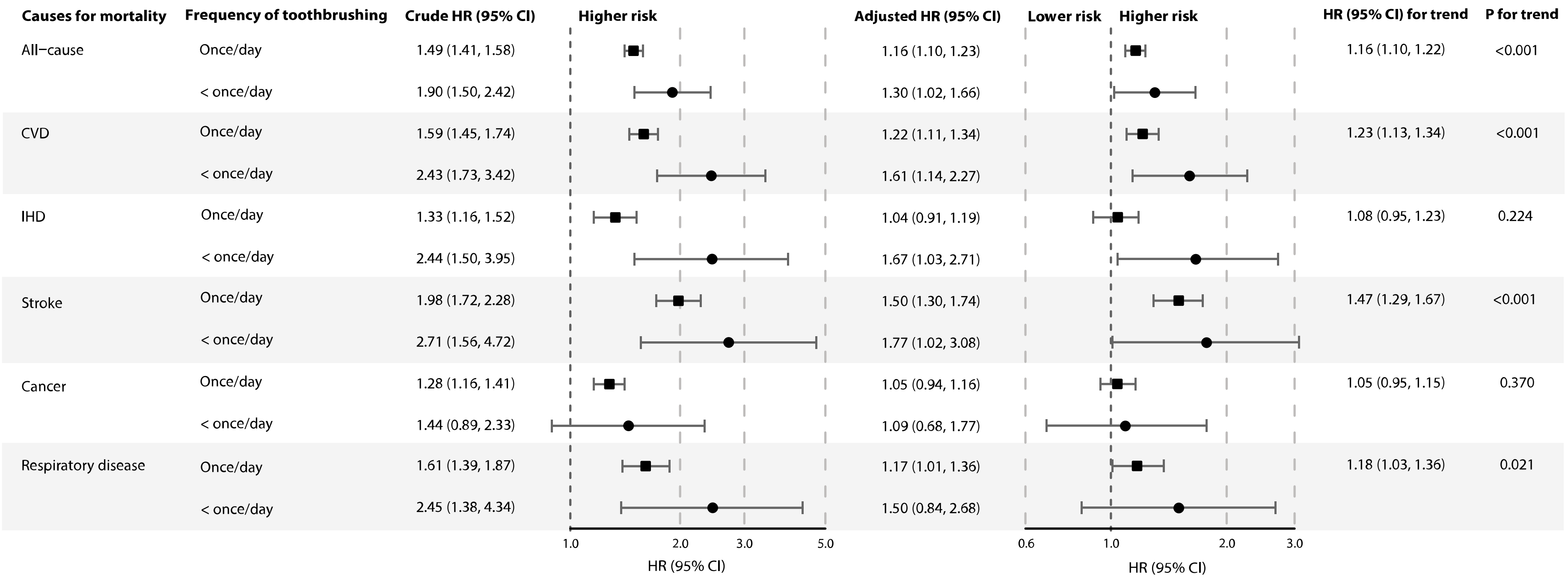

Supplementary Figure 6. Associations of frequency of toothbrushing (versus ≥twice/day) with all-cause and cause-specific mortality in participants of the Guangzhou Biobank Cohort Study recruited from September 2003 to January 2008 and followed up until April 2021, excluding those reported all-cause deaths within the first 3 years. Forest plot showing HRs (log scale) in circles and 95% CI (horizontal line). HRs were unadjusted in crude models, and were adjusted for sex, age, education level, occupation, household income, smoking status, alcohol use, physical activity, and self-rated health in multivariable-adjusted models.

CVD = cardiovascular disease, IHD = ischemic heart disease, HR = hazard ratio, CI = confidence interval.

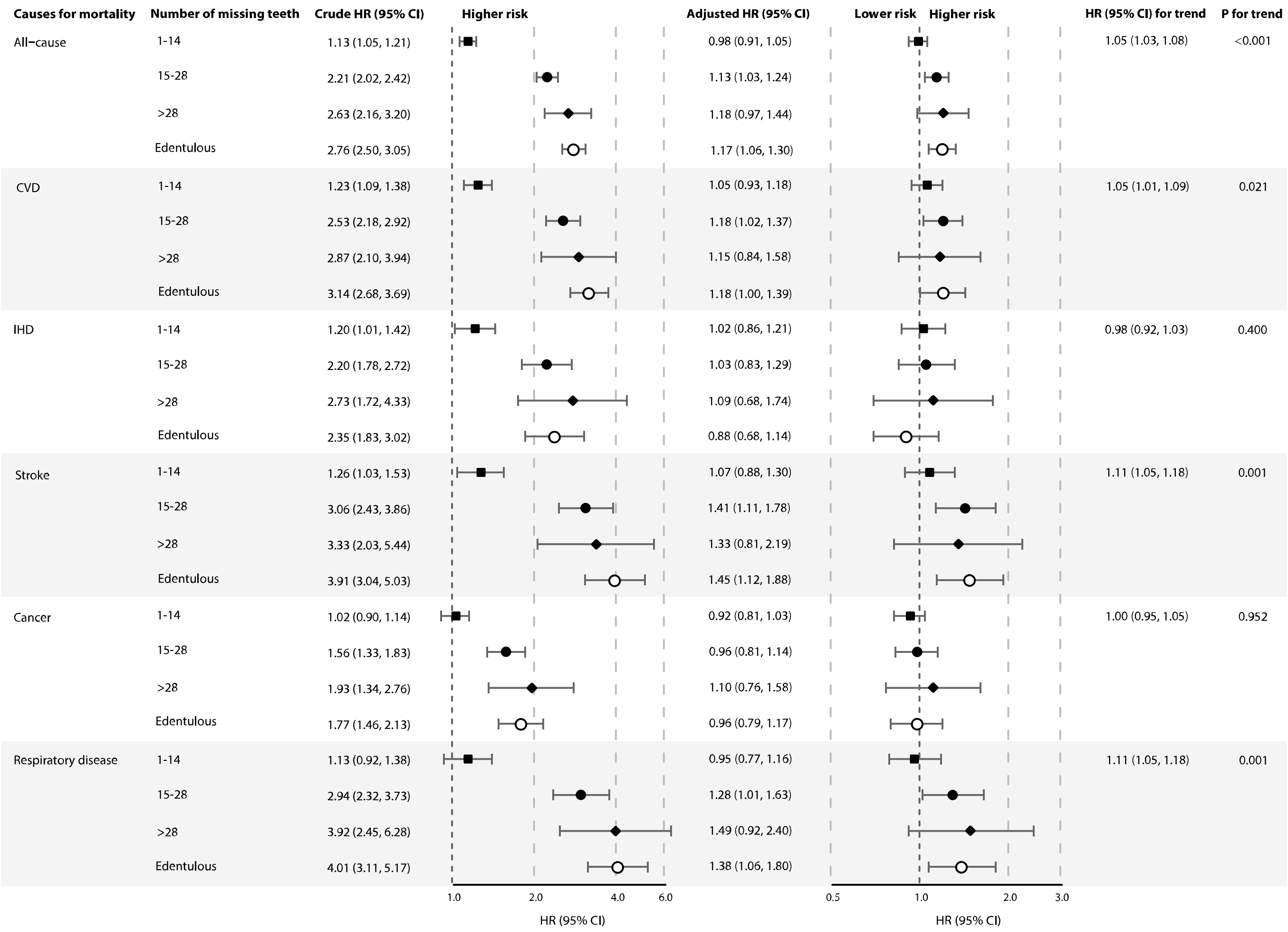

Supplementary Figure 7. Associations of number of missing teeth (versus 0) with all-cause and cause-specific mortality on participants of the Guangzhou Biobank Cohort

Study recruited from September 2003 to January 2008 and followed up until April 2021, excluding those reported all-cause deaths within the first 3 years. Forest plot showing HRs (log scale) in circles and 95% CI (horizontal line). HRs were unadjusted in crude models, and were adjusted for sex, age, education level, occupation, household income, smoking status, alcohol use, physical activity, self-rated health, and diabetes in multivariable-adjusted models.

CVD = cardiovascular disease, IHD = ischemic heart disease, HR = hazard ratio, CI = confidence interval.

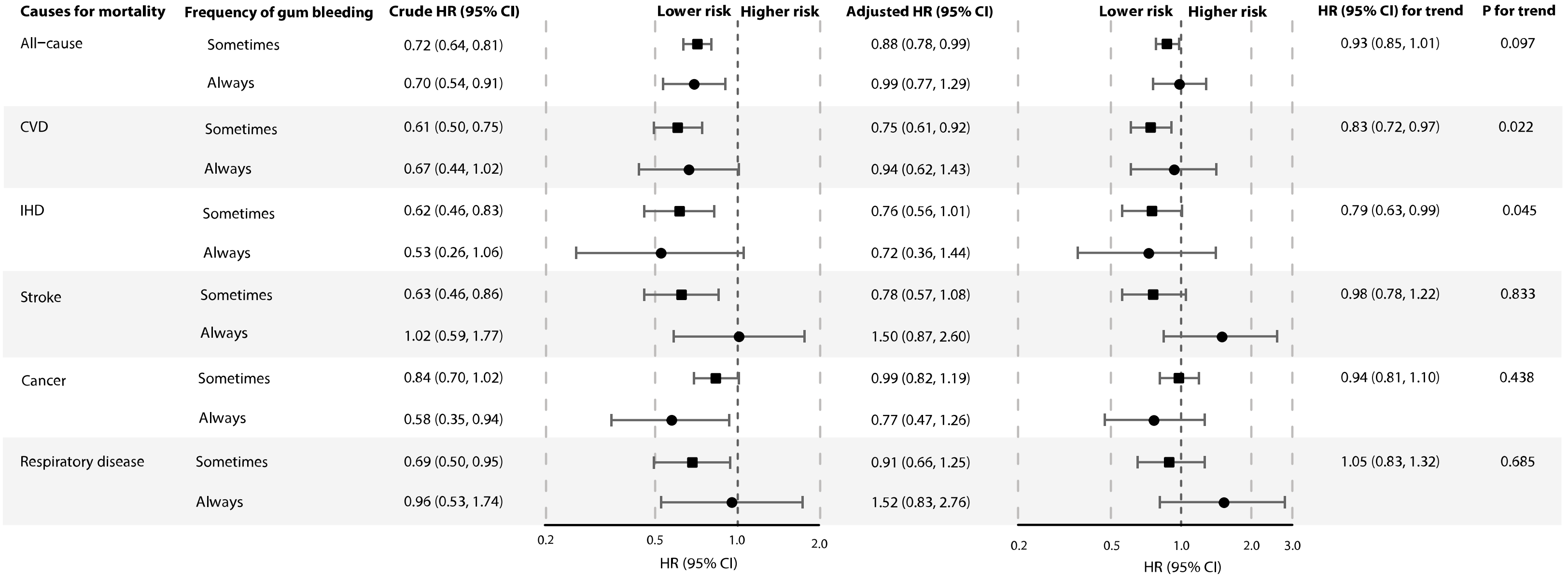

Supplementary Figure 8. Associations of gum bleeding (versus rarely/occasionally) with all-cause and cause-specific mortality on participants of the Guangzhou Biobank Cohort Study recruited from September 2003 to January 2008 and followed up until April 2021, excluding those reported all-cause deaths within the first 3 years. Forest plot showing HRs (log scale) in circles and 95% CI (horizontal line). HRs were unadjusted in crude models, and were adjusted for sex, age, education level, occupation, household income, smoking status, alcohol use, physical activity, and self-rated health in multivariable-adjusted models.

CVD = cardiovascular disease, IHD = ischemic heart disease, HR = hazard ratio, CI = confidence interval.

Supplementary Table 4 Association of frequency of toothbrushing (versus ≥twice/day) with all-cause and cause-specific mortality on participants of the Guangzhou Biobank Cohort Study recruited from September 2003 to January 2008 and followed up until April 2021, excluding those reported poor self-rated health status at baseline

**Causes of mortality**	**Frequency of toothbrushing**	**Crude HR (95% CI)**	**Adjusted HR (95% CI)**	**HR (95% CI) for trend**	**P for trend**
**All-cause**	Once/day	**1.47 (1.38, 1.56)*** **	**1.14 (1.07, 1.22)*** **	**1.14 (1.07, 1.20)**	**<0.001**
	<once/day	**1.84 (1.43, 2.38)*** **	1.20 (0.93, 1.55)		
**CVD**	Once/day	**1.62 (1.47, 1.79)*** **	**1.25 (1.13, 1.39)*** **	**1.25 (1.14, 1.37)**	**<0.001**
	<once/day	**2.45 (1.70, 3.52)*** **	**1.51 (1.05, 2.17)* **		
**IHD**	Once/day	**1.35 (1.16, 1.57)*** **	1.07 (0.91, 1.24)	1.10 (0.96, 1.27)	0.167
	<once/day	**2.49 (1.49, 4.17)** **	1.58 (0.94, 2.66)		
**Stroke**	Once/day	**2.01 (1.72, 2.35)*** **	**1.52 (1.29, 1.78)*** **	**1.46 (1.26, 1.69)**	**<0.001**
	<once/day	**2.65 (1.45, 4.83)** **	1.59 (0.87, 2.91)		
**Cancer**	Once/day	**1.23 (1.11, 1.37)*** **	1.01 (0.91, 1.13)	1.01 (0.91, 1.12)	0.840
	<once/day	1.38 (0.84, 2.25)	1.03 (0.63, 1.69)		

**Respiratory disease**	Once/day	**1.58 (1.34, 1.88)***	1.15 (0.97, 1.37)	1.15 (0.98, 1.35)	0.081
	<once/day	**2.33 (1.24, 4.38)**	1.33 (0.70, 2.50)		

HRs were unadjusted in crude models, and were adjusted for sex, age, education level, occupation, household income, smoking status, alcohol use, and physical activity in multivariable-adjusted models. Frequency of toothbrushing of ≥twice/day was selected as reference group.

HR = hazard ratio, CI = confidence interval, CVD = cardiovascular disease, IHD = ischemic heart disease.

* P<0.05, ** P<0.01, *** P<0.001.

Supplementary Table 5 Association of number of missing teeth (versus 0) with all-cause and cause-specific mortality on participants of the Guangzhou Biobank Cohort Study recruited from September 2003 to January 2008 and followed up until April 2021, excluding those reported poor self-rated health status at baseline

Causes of mortality	Number of missing teeth	Crude HR (95% CI)	Adjusted HR (95% CI)	HR (95% CI) for trend	P for trend
**All-cause**	1-14	**1.16 (1.07, 1.25)***	1.01 (0.93, 1.09)	**1.06 (1.03, 1.08)**	**<0.001**
	15-28	**2.26 (2.04, 2.49)***	**1.16 (1.05, 1.29)**		
	>28	**3.05 (2.48, 3.74)***	**1.44 (1.17, 1.77)**		

	Edentulous	**2.76 (2.47, 3.08)***	**1.17 (1.05, 1.31)**		
**CVD**	1-14	**1.20 (1.05, 1.37)**	1.02 (0.90, 1.16)	**1.04 (1.01, 1.09)**	**0.045**
	15-28	**2.47 (2.10, 2.90)***	1.15 (0.98, 1.36)		
	>28	**3.52 (2.54, 4.87)***	**1.46 (1.05, 2.04)***		
	Edentulous	**3.10 (2.60, 3.70)***	1.13 (0.94, 1.36)		
**IHD**	1-14	1.19 (0.98, 1.43)	1.00 (0.83, 1.21)	0.97 (0.91, 1.04)	0.355
	15-28	**2.12 (1.66, 2.70)***	0.99 (0.77, 1.26)		
	>28	**3.22 (1.98, 5.25)***	1.35 (0.82, 2.21)		
	Edentulous	**2.35 (1.78, 3.10)***	0.84 (0.63, 1.12)		
**Stroke**	1-14	**1.25 (1.01, 1.55)***	1.07 (0.86, 1.32)	**1.09 (1.02, 1.16)**	**0.015**
	15-28	**2.94 (2.27, 3.80)***	**1.36 (1.05, 1.77)***		
	>28	**3.78 (2.24, 6.39)***	1.59 (0.93, 2.70)		
	Edentulous	**3.55 (2.67, 4.71)***	1.31 (0.97, 1.76)		

**Cancer**	1-14	1.06 (0..93, 1.20)	0.95 (0.84, 1.08)	1.01 (0.96, 1.06)	0.612
	15-28	**1.65 (1.39, 1.96)*****	1.02 (0.86, 1.22)		
	>28	**2.19 (1.51, 3.18)*****	1.28 (0.88, 1.87)		
	Edentulous	**1.84 (1.51, 2.24)*****	1.00 (0.81, 1.23)		
**Respiratory disease**	1-14	1.21 (0.96, 1.52)	1.03 (0.82, 1.30)	**1.11 (1.03, 1.19)**	**0.004**
	15-28	**3.10 (2.37, 4.05)*****	**1.41 (1.07, 1.85)***		
	>28	**3.25 (1.79, 5.91)*****	1.28 (0.70, 2.35)		
	Edentulous	**4.04 (3.03, 5.40)*****	**1.41 (1.04, 1.90)***		

HRs were unadjusted in crude models, and were adjusted for sex, age, education level, occupation, household income, smoking status, alcohol use, physical activity, and diabetes in multivariable-adjusted models. Number of missing teeth of 0 was selected as reference group.

HR = hazard ratio, CI = confidence interval, CVD = cardiovascular disease, IHD = ischemic heart disease.

* P<0.05, ** P<0.01, *** P<0.001.

Supplementary Table 6 Association of frequency of gum bleeding (versus occasionally/rarely) with all-cause and cause-specific mortality on participants of the Guangzhou Biobank Cohort Study recruited from September 2003 to January 2008 and followed up until April 2021, excluding those reported poor self-rated health status at baseline

Causes of mortality	Frequency of toothbrushing	Crude HR (95% CI)	Adjusted HR (95% CI)	HR (95% CI) for trend	P for trend
**All-cause**	Sometimes	**0.70 (0.62, 0.80)*** **	**0.85 (0.75, 0.97)***	**0.90 (0.81, 0.99)**	**0.040**
	Always	**0.66 (0.49, 0.90)****	0.95 (0.70, 1.29)		
**CVD**	Sometimes	**0.55 (0.44, 0.70)*****	**0.69 (0.54, 0.87)****	**0.77 (0.64, 0.92)**	**0.005**
	Always	**0.58 (0.35, 0.99)***	0.84 (0.50, 1.42)		
**IHD**	Sometimes	**0.54 (0.38, 0.77)****	**0.67 (0.47, 0.96)***	**0.69 (0.51, 0.92)**	**0.013**
	Always	**0.37 (0.14, 0.98)***	0.52 (0.19, 1.39)		
**Stroke**	Sometimes	**0.58 (0.40, 0.85)****	0.73 (0.50, 1.06)	0.92 (0.71, 1.21)	0.566
	Always	**0.98 (0.51, 1.89)**	1.46 (0.75, 2.82)		
**Cancer**	Sometimes	**0.81 (0.66, 0.99)***	0.94 (0.76, 1.16)	0.90 (0.76, 1.06)	0.200
	Always	**0.49 (0.27, 0.89)***	0.67 (0.37, 1.21)		

**Respiratory disease**	Sometimes	0.80 (0.57, 1.12)	1.04 (0.74, 1.46)	1.13 (0.88, 1.46)	0.347
	Always	0.99 (0.49, 2.00)	1.57 (0.78, 3.17)		

HRs were unadjusted in crude models, and were adjusted for sex, age, education level, occupation, household income, smoking status, alcohol use, physical activity, and frequency of toothbrushing in multivariable-adjusted models. Frequency of gum bleeding of occasionally/rarely was selected as reference group.

HR = hazard ratio, CI = confidence interval, CVD = cardiovascular disease, IHD = ischemic heart disease.

* P<0.05, ** P<0.01, *** P<0.001.

Causes for mortality	Age subgroup	Frequency of toothbrushing	Adjusted HR (95% CI)	P for interaction	HR (95% CI) for trend	P for trend
All−cause	<65	Once/day	1.08 (0.97, 1.19)	0.632	1.08 (0.98, 1.18)	0.131
		< once/day	1.16 (0.67, 2.00)			
	≥65	Once/day	1.20 (1.12, 1.28)		1.20 (1.13, 1.28)	<0.001
		< once/day	1.49 (1.15, 1.94)			
CVD	<65	Once/day	1.02 (0.86, 1.22)	0.398	1.05 (0.88, 1.24)	0.603
		< once/day	1.54 (0.69, 3.46)			
	≥65	Once/day	1.31 (1.18, 1.45)		1.31 (1.19, 1.44)	<0.001
		< once/day	1.80 (1.23, 2.62)			
IHD	<65	Once/day	0.90 (0.69, 1.17)	0.427	0.99 (0.77, 1.28)	0.964
		< once/day	2.87 (1.17, 7.03)			
	≥65	Once/day	1.12 (0.96, 1.31)		1.14 (0.99, 1.32)	0.066
		< once/day	1.59 (0.90, 2.83)			
Stroke	<65	Once/day	1.31 (0.98, 1.74)	0.510	1.28 (0.96, 1.71)	0.087
		< once/day	0.72 (0.10, 5.14)			
	≥65	Once/day	1.54 (1.31, 1.82)		1.53 (1.32, 1.78)	<0.001
		< once/day	2.17 (1.22, 3.88)			
Cancer	<65	Once/day	1.02 (0.88, 1.19)	0.969	1.03 (0.89, 1.20)	0.651
		< once/day	1.31 (0.58, 2.92)			
	≥65	Once/day	1.04 (0.92, 1.18)		1.05 (0.93, 1.18)	0.439
		< once/day	1.21 (0.71, 2.06)			
Respiratory disease	<65	Once/day	1.14 (0.81, 1.59)	0.886	1.12 (0.81, 1.55)	0.478
		< once/day	1.03 (0.14, 7.40)			
	≥65	Once/day	1.19 (1.01, 1.40)		1.22 (1.04, 1.43)	0.013
		< once/day	1.89 (1.04, 3.46)			

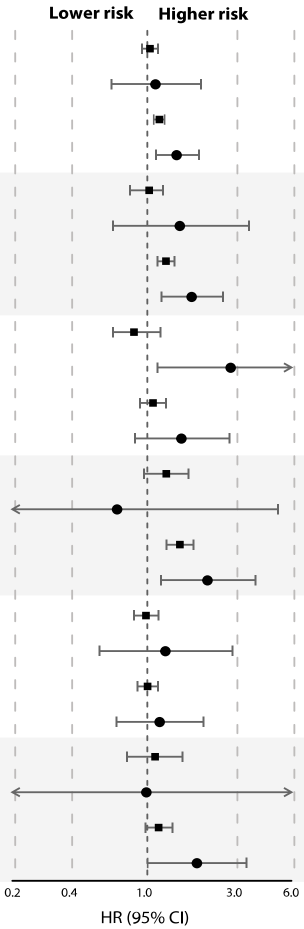

Supplementary Figure 9. Associations of frequency of toothbrushing (versus ≥twice/day) with all-cause and cause-specific mortality in 28,006 participants of the

Guangzhou Biobank Cohort Study recruited from September 2003 to January 2008 and followed up until April 2021, stratifying by age subgroup. Forest plot showing HRs (log scale) and 95% CI (horizontal line). HRs were adjusted for sex, education level, occupation, household income, smoking status, alcohol use, physical activity, and self-rated health in multivariable-adjusted models.

CVD = cardiovascular disease, IHD = ischemic heart disease, HR = hazard ratio, CI = confidence interval.

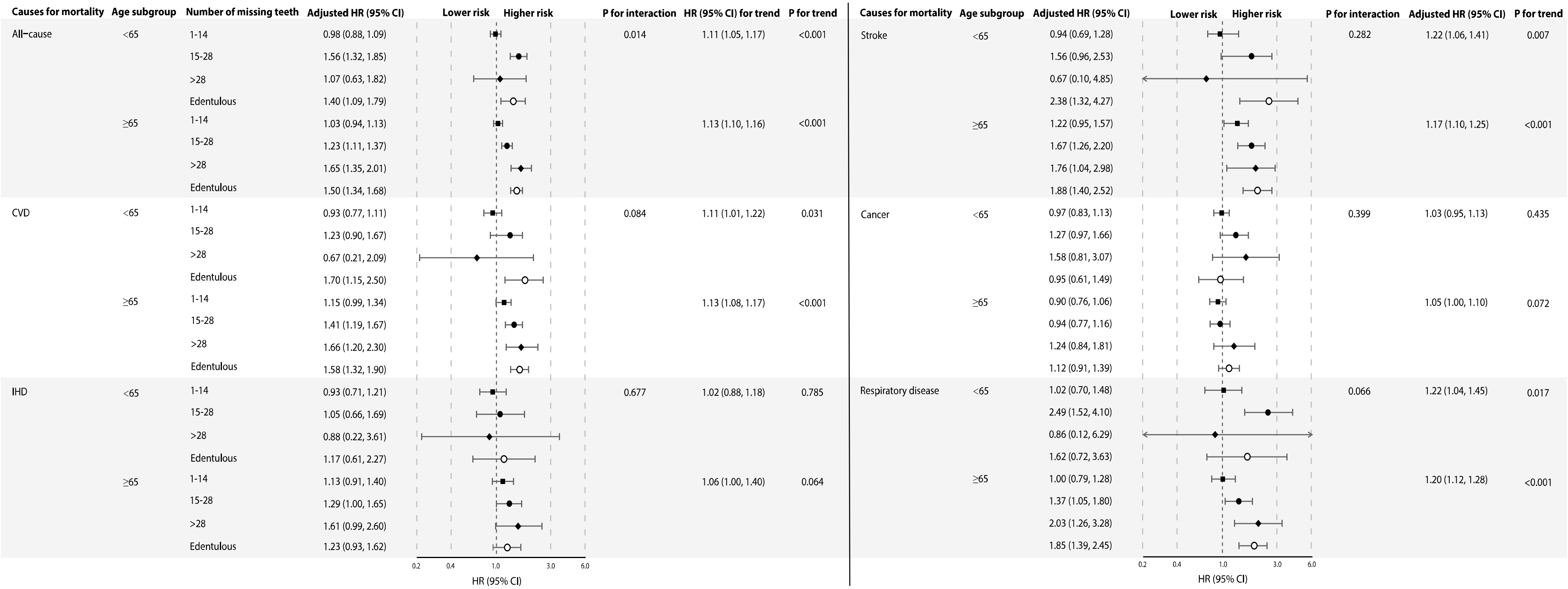

Causes for mortality	Age subgroup	Number of missing teeth	Adjusted HR (95% CI)	P for interaction	HR (95% CI) for trend	P for trend
All-cause	<65	1-14	0.98 (0.88, 1.09)	0.014	1.11 (1.05, 1.17)	<0.001
		15-28	1.56 (1.32, 1.85)			
		>28	1.07 (0.63, 1.82)			
		Edentulous	1.40 (1.09, 1.79)			
	≥65	1-14	1.03 (0.94, 1.13)		1.13 (1.10, 1.16)	<0.001
		15-28	1.23 (1.11, 1.37)			
		>28	1.65 (1.35, 2.01)			
		Edentulous	1.50 (1.34, 1.68)			
CVD	<65	1-14	0.93 (0.77, 1.11)	0.084	1.11 (1.01, 1.22)	0.031
		15-28	1.23 (0.90, 1.67)			
		>28	0.67 (0.21, 2.09)			
		Edentulous	1.70 (1.15, 2.50)			
	≥65	1-14	1.15 (0.99, 1.34)		1.13 (1.08, 1.17)	<0.001
		15-28	1.41 (1.19, 1.67)			
		>28	1.66 (1.20, 2.30)			
		Edentulous	1.58 (1.32, 1.90)			
IHD	<65	1-14	0.93 (0.71, 1.21)	0.677	1.02 (0.88, 1.18)	0.785
		15-28	1.05 (0.66, 1.69)			
		>28	0.88 (0.22, 3.61)			
		Edentulous	1.17 (0.61, 2.27)			
	≥65	1-14	1.13 (0.91, 1.40)		1.06 (1.00, 1.40)	0.064
		15-28	1.29 (1.00, 1.65)			
		>28	1.61 (0.99, 2.60)			
		Edentulous	1.23 (0.93, 1.62)			

Causes for mortality	Age subgroup	Adjusted HR (95% CI)	P for interaction	Adjusted HR (95% CI)	P for trend
Stroke	<65	0.94 (0.69, 1.28)	0.282	1.22 (1.06, 1.41)	0.007
		1.56 (0.96, 2.53)			
		0.67 (0.10, 4.85)			
		2.38 (1.32, 4.27)			
	≥65	1.22 (0.95, 1.57)		1.17 (1.10, 1.25)	<0.001
		1.67 (1.26, 2.20)			
		1.76 (1.04, 2.98)			
		1.88 (1.40, 2.52)			
Cancer	<65	0.97 (0.83, 1.13)	0.399	1.03 (0.95, 1.13)	0.435
		1.27 (0.97, 1.66)			
		1.58 (0.81, 3.07)			
		0.95 (0.61, 1.49)			
	≥65	0.90 (0.76, 1.06)		1.05 (1.00, 1.10)	0.072
		0.94 (0.77, 1.16)			
		1.24 (0.84, 1.81)			
		1.12 (0.91, 1.39)			
Respiratory disease	<65	1.02 (0.70, 1.48)	0.066	1.22 (1.04, 1.45)	0.017
		2.49 (1.52, 4.10)			
		0.86 (0.12, 6.29)			
		1.62 (0.72, 3.63)			
	≥65	1.00 (0.79, 1.28)		1.20 (1.12, 1.28)	<0.001
		1.37 (1.05, 1.80)			
		2.03 (1.26, 3.28)			
		1.85 (1.39, 2.45)			

Supplementary Figure 10. Associations of number of missing teeth (versus 0) with all-cause and cause-specific mortality in 28,006 participants of the Guangzhou Biobank Cohort Study recruited from September 2003 to January 2008 and followed up until April 2021, stratifying by age subgroup. Forest plot showing HRs (log scale) and 95% CI (horizontal line). HRs were adjusted for sex, education level, occupation, household income, smoking status, alcohol use, physical activity, self-rated health, and diabetes in multivariable-adjusted models.

CVD = cardiovascular disease, IHD = ischemic heart disease, HR = hazard ratio, CI = confidence interval.

Causes for mortality	Age group	Frequency of gum bleeding	Adjusted HR (95% CI)	P for interaction	HR (95% CI) for trend	P for trend
All−cause	<65	Somtimes	1.04 (0.88, 1.22)	0.003	1.02 (0.90, 1.15)	0.785
		Always	0.99 (0.70, 1.41)			
	≥65	Somtimes	0.69 (0.59, 0.80)		0.77 (0.68, 0.87)	<0.001
		Always	0.86 (0.60, 1.23)			
CVD	<65	Somtimes	0.98 (0.73, 1.32)	0.036	0.98 (0.73, 1.32)	0.886
		Always	0.94 (0.50, 1.77)			
	≥65	Somtimes	0.56 (0.43, 0.73)		0.56 (0.43, 0.73)	<0.001
		Always	0.80 (0.46, 1.42)			
IHD	<65	Somtimes	0.99 (0.64, 1.54)	0.226	0.95 (0.68, 1.32)	0.752
		Always	0.80 (0.30, 2.16)			
	≥65	Somtimes	0.58 (0.39, 0.85)		0.64 (0.46, 0.87)	0.005
		Always	0.59 (0.22, 1.57)			
Stroke	<65	Somtimes	0.97 (0.59, 1.59)	0.340	1.06 (0.75, 1.50)	0.746
		Always	1.32 (0.54, 3.21)			
	≥65	Somtimes	0.59 (0.39, 0.89)		0.81 (0.60, 1.08)	0.153
		Always	1.37 (0.68, 2.76)			
Cancer	<65	Somtimes	1.17 (0.93, 1.48)	0.022	1.07 (0.89, 1.28)	0.466
		Always	0.92 (0.53, 1.59)			
	≥65	Somtimes	0.71 (0.53, 0.95)		0.72 (0.56, 0.92)	0.008
		Always	0.52 (0.22, 1.26)			
Respiratory disease	<65	Somtimes	0.97 (0.53, 1.74)	0.870	1.10 (0.74, 1.65)	0.634
		Always	1.52 (0.56, 4.13)			
	≥65	Somtimes	0.79 (0.55, 1.14)		0.93 (0.71, 1.23)	0.624
		Always	1.38 (0.66, 2.92)			

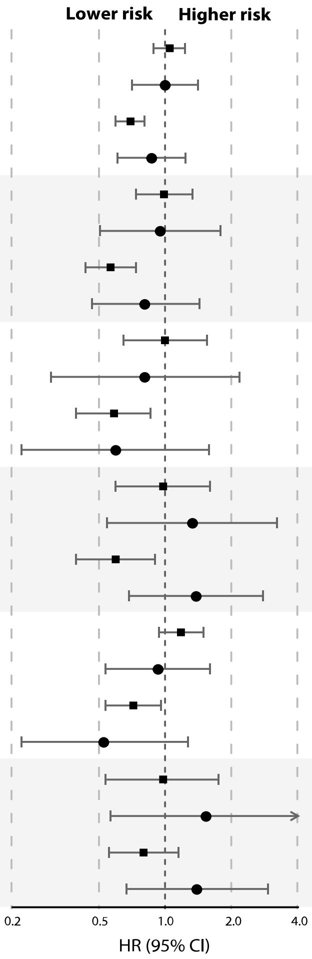

Supplementary Figure 11. Associations of frequency of gum bleeding (versus sometimes/often) with all-cause and cause-specific mortality in 28,006 participants of the

Guangzhou Biobank Cohort Study recruited from September 2003 to January 2008 and followed up until April 2021, stratifying by age subgroup. Forest plot showing HRs (log scale) and 95% CI (horizontal line). HRs were adjusted for sex, education level, occupation, household income, smoking status, alcohol use, physical activity, and self-rated health in multivariable-adjusted models.

CVD = cardiovascular disease, IHD = ischemic heart disease, HR = hazard ratio, CI = confidence interval.

Supplementary Table 7 Associations of frequency of toothbrushing (versus ≥twice/day) with all-cause and cause-specific mortality in 28,006 participants of the Guangzhou Biobank Cohort Study recruited from September 2003 to January 2008 and followed up until April 2021, stratifying by education level

Causes of mortality	Education level	Frequency of toothbrushing	Adjusted HR (95% CI)	P for interaction	HR (95% CI) for trend	P for trend
All-cause	Primary or below	Once/day	**1.19 (1.11, 1.28)*** **	0.280	**1.19 (1.11, 1.27)**	**<0.001**
		≤once/day	**1.39 (1.04, 1.85)***			
	Middle school and higher	Once/day	**1.11 (1.02, 1.21)***		**1.10 (1.01, 1.19)**	**0.022**
		≤once/day	1.05 (0.69, 1.61)			
CVD	Primary or below	Once/day	**1.26 (1.12, 1.41)*** **	0.369	**1.28 (1.15, 1.42)**	**<0.001**

		≤once/day	**1.81 (1.22, 2.68)****			
	Middle school and higher	Once/day	**1.17 (1.02, 1.35)***		**1.15 (1.01, 1.31)**	**0.042**
		≤once/day	1.03 (0.51, 2.08)			
IHD	Primary or below	Once/day	1.15 (0.97, 1.38)	0.226	**1.21 (1.03, 1.42)**	**0.021**
		≤once/day	**2.04 (1.16, 3.56)***			
	Middle school and higher	Once/day	0.94 (0.77, 1.16)		0.95 (0.78, 1.16)	0.606
		≤once/day	0.98 (0.37, 2.65)			
Stroke	Primary or below	Once/day	**1.40 (1.17, 1.68)*****	0.750	**1.38 (1.17, 1.63)**	**<0.001**
		≤once/day	1.68 (0.86, 3.28)			
	Middle school and higher	Once/day	**1.57 (1.23, 1.97)*****		**1.52 (1.23, 1.87)**	**<0.001**
		≤once/day	1.62 (0.60, 4.38)			
Cancer	Primary or below	Once/day	1.11 (0.97, 1.27)	0.156	1.10 (0.97, 1.25)	0.128
		≤once/day	1.10 (0.61, 2.01)			

	Middle school and higher	Once/day	0.95 (0.83, 1.10)		0.97 (0.85, 1.12)	0.703
		≤once/day	1.27 (0.66, 2.45)			
Respiratory disease	Primary or below	Once/day	1.11 (0.91, 1.36)	0.607	1.14 (0.95, 1.38)	0.153
		≤once/day	1.66 (0.82, 3.37)			
	Middle school and higher	Once/day	1.22 (0.97, 1.52)		1.19 (0.96, 1.46)	0.109
		≤once/day	1.08 (0.40, 2.95)			

HRs were adjusted for sex, age, occupation, household income, smoking status, alcohol use, physical activity, and self-rated health in multivariable-adjusted models.

Frequency of toothbrushing of ≥twice/day was selected as the reference group.

CVD = cardiovascular disease, IHD = ischemic heart disease, HR = hazard ratio, CI = confidence interval

* P<0.05, ** P<0.01, *** P<0.001.

Supplementary Table 8 Associations of number of missing teeth (versus 0) with all-cause and cause-specific mortality in 28,006 participants of the Guangzhou Biobank Cohort Study recruited from September 2003 to January 2008 and followed up until April 2021, stratifying by education level

Causes of mortality	Education level	Number of missing teeth	Adjusted HR (95% CI)	P for interaction	HR (95% CI) for trend	P for trend
All-cause	Primary or below	1-14	1.04 (0.94, 1.15)	**0.034**	**1.08 (1.04, 1.11)**	**<0.001**
		15-28	1.12 (0.99, 1.26)			
		>28	**1.39 (1.11, 1.74)****			
		Edentulous	**1.31 (1.15, 1.49)*****			
	Middle school and higher	1-14	0.91 (0.83, 1.00)		1.04 (1.00, 1.08)	0.057
		15-28	**1.18 (1.03, 1.34)***			
		>28	1.19 (0.86, 1.65)			
		Edentulous	1.06 (0.90, 1.25)			
CVD	Primary or below	1-14	1.12 (0.95, 1.32)	**0.003**	**1.06 (1.02, 1.11)**	**0.009**
		15-28	1.08 (0.89, 1.31)			

		>28	1.27 (0.88, 1.84)			
		Edentulous	**1.33 (1.08, 1.63)****			
	Middle school and higher	1-14	0.91 (0.78, 1.07)		1.03 (0.96, 1.09)	0.419
		15-28	**1.33 (1.07, 1.65)***			
		>28	1.08 (0.61, 1.90)			
		Edentulous	0.95 (0.72, 1.25)			
IHD	Primary or below	1-14	1.10 (0.86, 1.40)	0.114	1.01 (0.94, 1.09)	0.771
		15-28	0.98 (0.73, 1.32)			
		>28	1.29 (0.74, 2.25)			
		Edentulous	1.08 (0.79, 1.48)			
	Middle school and higher	1-14	0.91 (0.73, 1.15)		0.94 (0.86, 1.04)	0.212
		15-28	1.14 (0.84, 1.55)			
		>28	1.07 (0.49, 2.31)			

		Edentulous	**0.64 (0.41, 0.99)***			
Stroke	Primary or below	1-14	1.26 (0.96, 1.64)	**0.011**	**1.11 (1.03, 1.19)**	**0.005**
		15-28	1.28 (0.94, 1.75)			
		>28	1.35 (0.74, 2.46)			
		Edentulous	**1.64 (1.19, 2.27)****			
	Middle school and higher	1-14	0.88 (0.66, 1.16)		1.10 (0.99, 1.22)	0.069
		15-28	**1.67 (1.18, 2.35)****			
		>28	1.23 (0.49, 3.07)			
		Edentulous	1.15 (0.74, 1.79)			
Cancer	Primary or below	1-14	0.96 (0.80, 1.14)	0.733	1.02 (0.96, 1.08)	0.458
		15-28	1.01 (0.81, 1.25)			
		>28	1.07 (0.69, 1.66)			
		Edentulous	1.06 (0.83, 1.36)			

	Middle school and higher	1-14	0.89 (0.77, 1.03)		1.00 (0.93, 1.07)	0.920
		15-28	0.93 (0.74, 1.18)			
		>28	1.52 (0.93, 2.50)			
		Edentulous	0.95 (0.71, 1.26)			
Respiratory disease	Primary or below	1-14	0.96 (0.72, 1.27)	0.266	1.08 (1.00, 1.17)	0.054
		15-28	1.10 (0.79, 1.52)			
		>28	1.50 (0.85, 2.66)			
		Edentulous	1.26 (0.90, 1.78)			
	Middle school and higher	1-14	0.98 (0.73, 1.31)		**1.16 (1.06, 1.27)**	**0.001**
		15-28	**1.62 (1.14, 2.30)****			
		>28	1.45 (0.65, 3.21)			
		Edentulous	**1.57 (1.05, 2.34)***			

HRs were adjusted for sex, age, occupation, household income, smoking status, alcohol use, physical activity, self-rated health, and diabetes in multivariable-adjusted models.

Frequency of toothbrushing of ≥twice/day was selected as the reference group.

CVD = cardiovascular disease, IHD = ischemic heart disease, HR = hazard ratio, CI = confidence interval

* P<0.05, ** P<0.01, *** P<0.001.

Supplementary Table 9 Associations of frequency of gum bleeding (versus occasionally/rarely) with all-cause and cause-specific mortality in 28,006 participants of the Guangzhou Biobank Cohort Study recruited from September 2003 to January 2008 and followed up until April 2021, stratifying by education level

Causes of mortality	Education level	Frequency of gum bleeding	Adjusted HR (95% CI)	P for interaction	HR (95% CI) for trend	P for trend
All-cause	Primary or below	Sometimes	**0.77 (0.66, 0.91)****	0.173	**0.87 (0.77, 0.98)**	**0.024**
		Always	1.00 (0.72, 1.40)			
	Middle school and higher	Sometimes	0.95 (0.82, 1.11)		0.96 (0.85, 1.08)	0.487
		Always	0.93 (0.63, 1.36)			
CVD	Primary or below	Sometimes	**0.73 (0.56, 0.95)***	0.783	0.84 (0.69, 1.02)	0.085

		Always	1.01 (0.59, 1.71)			
	Middle school and higher	Sometimes	0.76 (0.57, 1.02)		0.80 (0.64, 1.01)	0.060
		Always	0.77 (0.38, 1.54)			
IHD	Primary or below	Sometimes	0.87 (0.60, 1.27)	0.537	0.85 (0.63, 1.14)	0.276
		Always	0.65 (0.24, 1.74)			
	Middle school and higher	Sometimes	**0.63 (0.40, 0.99)***		0.72 (0.51, 1.02)	0.062
		Always	0.76 (0.28, 2.03)			
Stroke	Primary or below	Sometimes	0.57 (0.35, 0.91)	0.119	0.89 (0.65, 1.20)	0.435
		Always	1.69 (0.87, 3.27)			
	Middle school and higher	Sometimes	1.05 (0.69, 1.61)		1.05 (0.76, 1.45)	0.777
		Always	1.09 (0.40, 2.92)			
Cancer	Primary or below	Sometimes	0.77 (0.57, 1.04)	**0.049**	**0.76 (0.59, 0.96)**	**0.023**
		Always	0.53 (0.24, 1.19)			

	Middle school and higher	Sometimes	1.12 (0.89, 1.41)		1.07 (0.90, 1.28)	0.444
		Always	1.01 (0.57, 1.78)			
Respiratory disease	Primary or below	Sometimes	0.95 (0.62, 1.45)	0.165	1.21 (0.91, 1.60)	0.191
		Always	**2.17 (1.12, 4.21)***			
	Middle school and higher	Sometimes	0.88 (0.55, 1.40)		0.84 (0.57, 1.24)	0.380
		Always	0.60 (0.15, 2.40)			

HRs were adjusted for sex, age, occupation, household income, smoking status, alcohol use, physical activity, and self-rated health in multivariable-adjusted models.

Frequency of gum bleeding of occasionally/rarely was selected as the reference group.

CVD = cardiovascular disease, IHD = ischemic heart disease, HR = hazard ratio, CI = confidence interval

* P<0.05, ** P<0.01, *** P<0.001.

Supplementary Table 10 Proportions of the association of frequency of toothbrushing (versus ≥twice/day) and all-cause and cause-specific mortality attributable to total energy intake (kcal/day)

Causes for mortality	Frequency of toothbrushing	Model 1	Model 2	PERM%
**All-cause**	≤once/day	1.172 (1.108, 1.239)	1.171 (1.107, 1.237)	**0.6%**
**CVD**	≤once/day	1.244 (1.138, 1.360)	1.243 (1.137, 1.359)	**0.4%**
**IHD**	≤once/day	1.082 (0.946, 1.236)	1.083 (0.947, 1.237)	1.2%
**Stroke**	≤once/day	1.504 (1.304, 1.734)	1.499 (1.300, 1.728)	**1.0%**
**Cancer**	≤once/day	1.038 (0.942, 1.145)	1.039 (0.942, 1.146)	2.6%
**Respiratory disease**	≤once/day	1.207 (1.037, 1.404)	1.206 (1.037, 1.403)	**0.5%**

Model 1: adjusting for sex, age, education level, occupation, household income, smoking status, alcohol use, physical activity, and self-rated health status.

Model 2: adjusting for sex, age, education level, occupation, household income, smoking status, alcohol use, physical activity, self-rated health status, and total energy intake.

CVD = cardiovascular disease, IHD = ischemic heart disease.

Supplementary Table 11 Proportions of the association of frequency of toothbrushing (versus ≥twice/day) and all-cause and cause-specific mortality attributable to dietary habit

Causes for mortality	Frequency of toothbrushing	Model 1	Model 2	PERM%
**All-cause**	≤once/day	1.161 (1.099, 1.226)	1.163 (1.101, 1.228)	**1.2%**
**CVD**	≤once/day	1.235 (1.132, 1.348)	1.238 (1.134, 1.351)	**1.3%**
**IHD**	≤once/day	1.074 (0.942, 1.225)	1.079 (0.946, 1.230)	6.8%
**Stroke**	≤once/day	1.485 (1.292, 1.707)	1.486 (1.293, 1.709)	**0.2%**
**Cancer**	≤once/day	1.035 (0.940, 1.140)	1.038 (0.942, 1.142)	8.6%
**Respiratory disease**	≤once/day	1.172 (1.011, 1.360)	1.176 (1.014, 1.364)	**2.3%**

Model 1: adjusting for sex, age, education level, occupation, household income, smoking status, alcohol use, physical activity, and self-rated health status.

Model 2: adjusting for sex, age, education level, occupation, household income, smoking status, alcohol use, physical activity, self-rated health status, and dietary habit.

CVD = cardiovascular disease, IHD = ischemic heart disease.

Supplementary Table 12 Proportions of the association of number of missing teeth (versus0) and all-cause and cause-specific mortality attributable to total energy intake (kcal/day)

Causes for mortality	Number of missing teeth	Model 1	Model 2	PERM, %
**All-cause**	14-28	1.132 (1.034, 1.240)	1.132 (1.034, 1.240)	**0.0%**
	>28	1.304 (1.074, 1.582)	1.301 (1.072, 1.580)	**1.0%**
	Edentulous	1.217 (1.099, 1.346)	1.216 (1.099, 1.345)	**0.5%**
**CVD**	14-28	1.147 (0.991, 1.327)	1.146 (0.905, 1.142)	0.7%
	>28	1.162 (0.840, 1.608)	1.146 (0.991, 1.326)	1.2%
	Edentulous	1.184 (1.006, 1.392)	1.183 (1.005, 1.391)	**0.5%**
**IHD**	14-28	1.038 (0.838, 1.286)	1.038 (0.838, 1.286)	0.0%
	>28	1.122 (0.697, 1.807)	1.124 (0.698, 1.810)	1.6%

	Edentulous	0.916 (0.713, 1.178)	0.918 (0.698, 1.179)	2.4%
**Stroke**	14-28	1.359 (1.073, 1.722)	1.358 (1.072, 1.719)	**0.3%**
	>28	1.207 (0.705, 2.068)	1.197 (0.699, 2.050)	4.8%
	Edentulous	1.416 (1.091, 1.839)	1.411 (1.087, 1.832)	**1.2%**
**Cancer**	14-28	0.961 (0.818, 1.129)	0.961 (0.818, 1.129)	0.0%
	>28	1.219 (0.864, 1.719)	1.221 (0.865, 1.225)	0.9%
	Edentulous	1.016 (0.844, 1.224)	1.017 (0.845, 1.225)	6.2%
**Respiratory disease**	14-28	1.374 (1.073, 1.758)	1.374 (1.074, 1.758)	**0.0%**
	>28	1.648 (1.009, 2.691)	1.647 (1.001, 2.689)	**0.2%**
	Edentulous	1.476 (1.129, 1.929)	1.475 (1.129, 1.928)	**0.2%**

Model 1: adjusting for sex, age, education level, occupation, household income, smoking status, alcohol use, physical activity, self-rated health status, and diabetes.

Model 2: adjusting for sex, age, education level, occupation, household income, smoking status, alcohol use, physical activity, self-rated health status,

diabetes, and total energy intake.

CVD = cardiovascular disease, IHD = ischemic heart disease.

Supplementary Table 13 Proportions of the association of number of missing teeth (versus 0) and all-cause and cause-specific mortality attributable to dietary habit

Causes for mortality	Number of missing teeth	Model 1	Model 2	PERM%
**All-cause**	14-28	1.125 (1.029, 1.230)	1.126 (1.030, 1.231)	**0.8%**
	>28	1.300 (1.082, 1.562)	1.301 (1.083, 1.563)	**0.3%**
	Edentulous	1.203 (1.089, 1.329)	1.203 (1.089, 1.329)	**0.0%**
**CVD**	14-28	1.154 (0.999, 1.332)	1.151 (0.997, 1.328)	1.9%
	>28	1.190 (0.876, 1.615)	1.179 (0.868, 1.601)	5.8%
	Edentulous	1.191 (1.015, 1.397)	1.191 (1.015, 1.398)	**0.0%**
**IHD**	14-28	1.036 (0.838, 1.281)	1.033 (0.835, 1.278)	8.4%

	>28	1.169 (0.748, 1.827)	1.167 (0.746, 1.824)	1.2%
	Edentulous	0.905 (0.706, 1.161)	0.908 (0.708, 1.165)	3.2%
**Stroke**	14-28	1.386 (1.100, 1.745)	1.067 (0.881, 1.293)	82.6%
	>28	1.272 (0.774, 2.089)	1.385 (0.768, 2.073)	41.5%
	Edentulous	1.450 (1.124, 1.872)	1.448 (1.122, 1.868)	**0.4%**
**Cancer**	14-28	0.958 (0.818, 1.122)	0.962 (0.821, 1.127)	9.5%
	>28	1.208 (0.870, 1.678)	1.219 (0.878, 1.692)	5.3%
	Edentulous	0.998 (0.830, 1.120)	0.999 (0.831, 1.200)	50.0%
**Respiratory disease**	14-28	1.316 (1.037, 1.670)	1.321 (1.041, 1.677)	**1.6%**
	>28	1.567 (0.990, 2.478)	1.577 (0.996, 2.496)	1.8%
	Edentulous	1.400 (1.079, 1.815)	1.398 (1.078, 1.813)	**0.5%**

Model 1: adjusting for sex, age, education level, occupation, household income, smoking status, alcohol use, physical activity, self-rated health status, and diabetes.

Model 2: adjusting for sex, age, education level, occupation, household income, smoking status, alcohol use, physical activity, self-rated health status, diabetes, and total energy intake.

CVD = cardiovascular disease, IHD = ischemic heart disease.
